# Hi-TrAC reveals division of labor of transcription factors in organizing chromatin loops

**DOI:** 10.1038/s41467-022-34276-8

**Published:** 2022-11-05

**Authors:** Shuai Liu, Yaqiang Cao, Kairong Cui, Qingsong Tang, Keji Zhao

**Affiliations:** Laboratory of Epigenome Biology, Systems Biology Center, Division of Intramural Research, National Heart, Lung, and Blood Institute, National Institutes of Health, Bethesda, MD USA

**Keywords:** Chromatin analysis, Chromatin structure, Epigenetics

## Abstract

The three-dimensional genomic structure plays a critical role in gene expression, cellular differentiation, and pathological conditions. It is pivotal to elucidate fine-scale chromatin architectures, especially interactions of regulatory elements, to understand the temporospatial regulation of gene expression. In this study, we report Hi-TrAC as a proximity ligation-free, robust, and sensitive technique to profile genome-wide chromatin interactions at high-resolution among regulatory elements. Hi-TrAC detects chromatin looping among accessible regions at single nucleosome resolution. With almost half-million identified loops, we reveal a comprehensive interaction network of regulatory elements across the genome. After integrating chromatin binding profiles of transcription factors, we discover that cohesin complex and CTCF are responsible for organizing long-range chromatin loops, related to domain formation; whereas ZNF143 and HCFC1 are involved in structuring short-range chromatin loops between regulatory elements, which directly regulate gene expression. Thus, we introduce a methodology to identify a delicate and comprehensive network of cis-regulatory elements, revealing the complexity and a division of labor of transcription factors in organizing chromatin loops for genome organization and gene expression.

## Abstract (web summary)

It is currently not clear how architectural proteins orchestrate chromatin looping at different scales of genome organisation. Here the authors report Hi-TrAC as a proximity ligation-free method to profile genome-wide chromatin interactions at single nucleosome resolution among regulatory elements.

## Introduction

The genome is organized into higher-order chromatin structures^1–6^. Each chromosome occupies a discrete territory in the nucleus^7^. Based on the spatial separation of active and inactive phases, the chromatin is partitioned into A and B compartments, respectively^7–9^. Self-associating chromatin assembles into ~1 Mb-sized topologically associating domains (TADs)^10–12^, which contain nested sub-TADs with the size of several hundred kb^13,14^. Chromatin domains are assembled by chromatin interaction loops, which are organized by CTCF and cohesin complex through a loop-extrusion process^15–26^. Interactions between transcriptional regulatory elements are important for orchestrating gene expression^27–43^. Knowledge of the detailed enhancer-promoter interaction network is important for understanding the fine-tuning of cell activities^44–46^. A sensitive and efficient technique is highly desired for elucidating genome-wide fine structures, particularly enhancer-promoter interactions. It is generally accepted that cohesin complex catalyzes chromatin folding into loops anchored by CTCF binding^18–23,43,47–50^, whereas several other chromatin factors, including ZNF143 and YY1, have been shown to facilitate chromatin loop formation^8,51–58^. However, it is not clear how these architectural proteins orchestrate chromatin looping at different scales of genome organization.

In this study, we developed a highly sensitive technique termed as Hi-TrAC (highly sensitive transposase-mediated analysis of chromatin) and applied it to profiling chromatin loops at Tn5 accessible chromatin regions in three different mouse and human cell types. By integrating the regulatory interaction network from the Hi-TrAC data with hundreds of transcription factor ChIP-seq data, we found that while CTCF and cohesin are involved in long-range chromatin interactions, HCFC1 and ZNF143 are involved in short-range chromatin loop formation.

## Results

### Technical improvements of Hi-TrAC

**Fig. 1:**
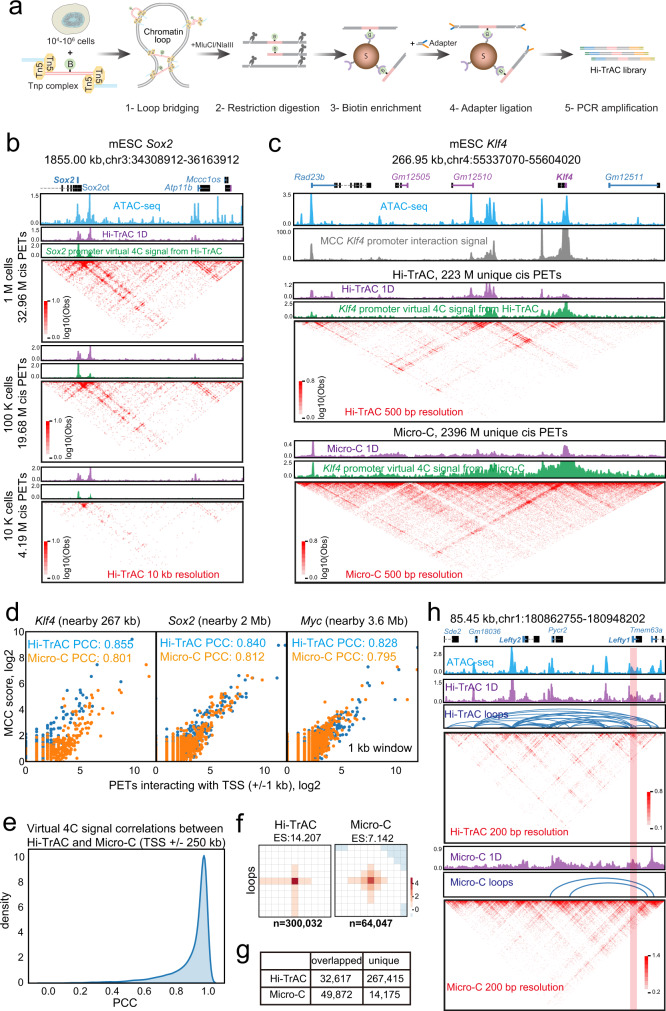
Mapping genome-wide regulatory interactions at high resolution by Hi-TrAC. **a** Experimental scheme of Hi-TrAC. Following bridging chromatin loops using the Tnp-biotinylated bivalent ME linker complex in formaldehyde fixed cells, the DNA is cleaved with restriction enzymes MluCI and NlaIII. The bridged genomic regions are enriched using streptavidin beads and PCR-amplified for sequencing after ligation of a universal adapter. **b** Hi-TrAC reproducibly detects interactions around the *Sox2* gene locus from 10^6^, 10^5^, and 10^4^ E14 mESCs. ATAC-seq data were obtained from GSM1830114^114^. Hi-TrAC virtual 4 C signals were generated by only keeping PETs interacting with the + /− 1 kb TSS region of *Sox2* gene and displayed as the piled up 1D signal. Interacting PETs were shown as dots below the 4C-like signals. The genomic annotations are shown on the top of the panel. The visualization was performed with cLoops2 plot module. **c** Comparison of interactions around *Klf4* gene locus from pooled MCC^62^, Hi-TrAC, and Micro-C^60^ data in mESCs. Only intra-chromosomal PETs from Hi-TrAC and Micro-C were used for comparisons. **d** Correlation analysis between interactions detected by MCC with the virtual 4 C signals from Hi-TrAC or Micro-C data around *Klf4*, *Sox2*, and *Myc* loci, with the viewpoint set as the + /− 1 kb of TSS. Source data are provided as a Source Data file. **e** Distribution of correlations between the virtual 4 C signals from Hi-TrAC and Micro-C around the promoter regions of all protein-coding genes. The promoter defined as a region + /− 1Kb upstream and downstream of a TSS was set as the viewpoint. Only PETs with any end located in the promoter region were kept, and a region of 250Kb upstream and downstream of TSSs was set as the comparing region. PCC stands for Pearson Correlation Coefficient. **f** Aggregation analysis of Hi-TrAC and Micro-C loops. Hi-TrAC loops were called by the cLoops2 callLoops module and requiring at least 20 PETs (Supplementary Data 2), and Micro-C loops were called by HiCCUPS. **g** Overlaps of Hi-TrAC and Micro-C loops. **h** Genome Browser snapshot of the *Lefty* locus, showing the distribution of ATAC-seq peaks and chromatin loops detected by Hi-TrAC and Micro-C as well as the interaction matrices at a 200 bp resolution.

Hi-TrAC originated from the Trac-looping (Transposase-mediated analysis of chromatin looping) method with substantial improvements^59^. Hi-TrAC takes advantage of DNA transposase Tn5’s ability of utilizing a specially designed bivalent linker to covalently bridge spatially proximal open chromatin regions, thus eliminating chromatin fragmentation and proximity ligation steps required for 3 C (chromosome conformation capture)-based techniques (Fig. 1a, Methods). We also designed a strategy to eliminate the rolling cycle amplification and dilute ligation in large volumes in Trac-looping, enabling us to reduce the starting material of 100 million cells to as few as 10 thousand cells and shorten library construction time from 7 days to 2 days.

### Hi-TrAC outperforms other techniques at detecting chromatin loops

We first benchmarked Hi-TrAC with the most advanced Hi-C variants Micro-C and Micro-Capture-C (MCC), which produce high-resolution chromatin structure maps^60–62^. Starting with 0.01, 0.1, and 1 million mouse embryonic stem cells (mESCs) (Supplementary Data 1), as exemplified by the *Sox2* gene locus, Hi-TrAC reproducibly detected similar chromatin interaction profiles (Fig. 1b). To compare with MCC data, we extracted all paired-end tags (PETs) linking to the promoters of *Klf4*, *Sox2*, and *Myc* genes from Hi-TrAC and Micro-C data and displayed them as virtual 4 C signals. Hi-TrAC detected chromatin interactions at these promoters were highly comparable to those detected by MCC and Micro-C, from both visual inspection and a quantitative correlation analysis (Fig. 1c, d). Moreover, the virtual 4 C signals from Hi-TrAC and Micro-C data were highly correlated at all gene promoters in the genome (Fig. 1e). With a higher signal-to-noise ratio, Hi-TrAC even showed higher correlation with MCC than Micro-C, and detected more details of fine chromatin structures (Fig. 1c, d and Supplementary Fig. 1a). These results indicate that Hi-TrAC is a robust and reliable technique for detecting interactions among chromatin regulatory regions. It is noteworthy that all these chromatin loops are among accessible chromatin regions, suggesting that chromatin accessibility is necessary for Hi-TrAC to detect chromatin loops.

Hi-TrAC performed a little more sensitively than Micro-C to detect interactions at different genomic distances (Supplementary Fig. 1b). We identified 300k significant chromatin loops from 223 million unique intra-chromosomal PETs (cis-PETs) in Hi-TrAC, compared to 64k loops from 2396 million unique cis-PETs in Micro-C (Supplementary Data 2). The aggregation analysis showed that Hi-TrAC loops displayed a higher enrichment score (ES) than Micro-C loops, indicating a higher signal-to-noise ratio in Hi-TrAC data (Fig. 1f). About 80% of Micro-C loops were identified by Hi-TrAC, whereas only 11% of Hi-TrAC loops were covered by Micro-C (Fig. 1g and Supplementary Fig. 1c). As exemplified by the looping profiles at loci of functionally important genes, including *Myc* (Supplementary Fig. 1a), *Lefty* (Fig. 1h), and *Nanog* **(**Supplementary Fig. 1d), more significant loops were identified by Hi-TrAC owing to its high signal-to-noise ratio. Especially at the promoters of *Lefty1* and *Nanog* genes, specific loops could only be identified by Hi-TrAC, but missed by Micro-C (Fig. 1h and Supplementary Fig. 1d). Generally, Micro-C unique loops are distal weaker loops (Supplementary Fig. 1e, f). The majority of both Micro-C (76%) and Hi-TrAC (92%) loop anchors were enriched at accessible chromatin regions as characterized by ATAC-seq peaks (Supplementary Fig. 1g) The rest did not show features of repressive chromatin (Supplementary Fig. 1h), suggesting that significant chromatin loops detected by these techniques were mainly between accessible regulatory elements. Together, these results indicate that Hi-TrAC is a sensitive method for detecting chromatin loops among active regulatory regions.

To further evaluate the performance of Hi-TrAC in elucidating chromatin structures, we applied Hi-TrAC to GM12878 cells, a human cell-line whose genome architecture had been extensively studied by various techniques (Supplementary Data 1). To obtain a comprehensive interaction map, we pooled the data from all experimental replicates, resulting in 822 million raw reads and 117 million unique intra-chromosomal PETs (Supplementary Data 1). As shown in the two-dimension (2D) heatmap at different resolutions, we compared the genome architecture map generated by Hi-TrAC with available maps built by in situ Hi-C^8^, CTCF^32^, and RAD21 ChIA-PET^51,63^, capture Hi-C^64^, H3K27ac HiChIP^65^, cohesin HiChIP^66^, and HiCAR^67^ (Supplementary Fig. 2). With relatively low sequencing depth, chromatin domain-like structures and loops could be clearly detected by Hi-TrAC at different resolutions; especially for identifying significant chromatin loops, Hi-TrAC data had a much higher signal-to-noise ratio than other methods; even at 200 bp resolution, the fine architectural details of super-enhancers could also be observed, which were not clear in the maps generated by other techniques (Supplementary Fig. 2). HiCAR is a recently developed proximity ligation-dependent technique that detects chromatin interactions by Tn5. By comparing the published HiCAR data with our Hi-TrAC data from GM12878 cells, we found that while the two methods performed similarly in detecting TADs-like structure (Supplementary Fig. 2b), Hi-TrAC outperformed HiCAR in the detected number of chromatin loops (91,042 vs. 48,515) and signal-to-noise ratio shown by enrichments scores (20.798 vs. 3.402) (Supplementary Figs. 3h and 5c).

To systematically compare the genome-wide highest resolution that these techniques can achieve, we calculated the coverage of PETs with different bin sizes. With the threshold of more than 50% of the PETs not being singleton PETs, only H3K27ac HiChIP could achieve a similar resolution to Hi-TrAC (Supplementary Fig. 3). We then performed sub-samplings of Hi-TrAC data to estimate the required sequencing depth for reaching the desired resolution. The analysis indicated that with 60 million intra-chromosomal PETs, about 300 million raw reads, genome-wide 1 kb resolution could be achieved (Supplementary Fig. 4a), and fine-scale architectures could also be identified at 200 bp resolution at a subset of genomic regions including super-enhancers (Supplementary Fig. 4b, c).

### Hi-TrAC detects comprehensive interaction network of cis-regulatory elements

**Fig. 2:**
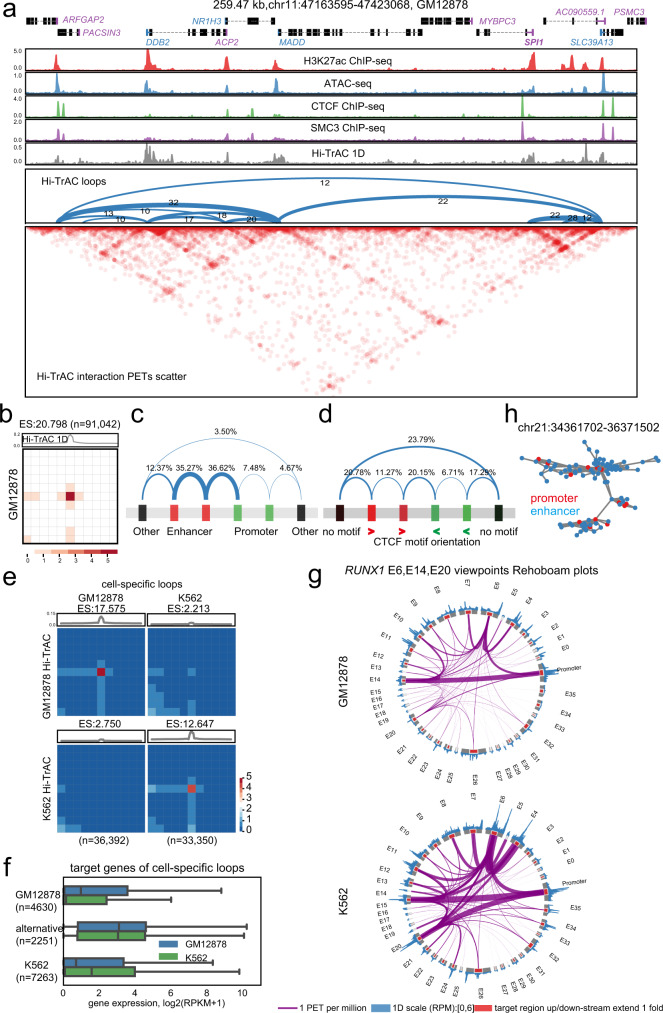
Chromatin looping networks constructed with Hi-TrAC data. **a** Genome Browser snapshot showing the chromatin loops detected by Hi-TrAC around the *SPI1* gene in GM12878 cells. Loops are shown as arches, and the numbers of PETs are also shown for each loop. Loops were called by the cLoops2 callLoops module, requiring at least ten supportive PETs. The interaction matrices are displayed at the bottom. **b** Aggregation analysis of 91,042 loops called from Hi-TrAC data in GM12878 (Supplementary Data 3). Interacting PETs in loops and their nearby regions (5-folds upstream and downstream of loop anchors) as matrices were averaged as the aggregated heatmap. ES stands for enrichment score, indicating the interaction signal enrichment compared to neighbor regions. The analysis was performed with cLoops2 agg module. **c** Summary of categories of GM12878 Hi-TrAC loops with regard to putative cis-regulatory elements, including enhancers, promoters and others. **d** Summary of categories of GM12878 Hi-TrAC loops with regard to the orientation of CTCF motifs at the two anchors of a loop. **e** Aggregation analysis of cell-specific loops in GM12878 and K562 (Supplementary Data 4). Differentially enriched loops were called with the cLoops2 callDiffLoops module. **f** The distribution of expression levels of the genes associated with cell-specific loops, and the genes with promoters looping with alternative enhancers between GM12878 and K562. The numbers of genes for each category were indicated. The box extends from the first quartile to the third quartile of the data, with a line at the median. The whiskers extend from the box by 1.5x the inter-quartile range. Flier points past the end of the whiskers were not shown. Source data are provided as a Source Data file. **g** Rehoboam plots showing the differences in promoter-enhancer interactions at *RUNX1* gene locus in GM12878 and K562 cells. Interactions from the viewpoints of enhancers E6, E14, and E20 are shown. Source data are provided as a Source Data file. **h** An example of the longest connected sub-network consisting of enhancers and promoters on Chromosome 21 in GM12878. Enhancers and promoters form complex connections as nature scale-free regulatory networks. Source data are provided as a Source Data file.

As expected, Hi-TrAC signals were highly concentrated at accessible regions (Supplementary Fig. 5a). There was a low correlation between Hi-TrAC interaction intensity with the accessibility of the loci within different genomic distance (Supplementary Fig. 5b), suggesting that Hi-TrAC detected interactions are specific spatial contacts. To explore how the fine-scale chromatin architectures are organized for individual cis-regulatory elements, we further analyzed the chromatin looping revealed by Hi-TrAC data. In GM12878 cells, as exemplified by the *SPI1* locus, which encodes the key lymphoid cell development-related ETS family transcription factor PU.1, we observed typical dot-to-dot chromatin loops pattern formed between distal and proximal regulatory elements (Fig. 2a**)**. Globally, with at least 10 PETs supporting a loop, we called 91,042 high-confidence loops in GM12878 cells (Fig. 2b and Supplementary Data 3), much more than other techniques (Supplementary Fig. 5c).

We compared various features of Hi-TrAC loops with those detected by other methods. Generally, Hi-TrAC loops were shorter in distance, with loop anchors concentrated at promoters and enhancers (Supplementary Fig. 5d–g). The majority of them were enhancer-promoter, and enhancer-enhancer loops (Fig. 2c). Compared to other datasets, the CTCF motif orientations at Hi-TrAC loop anchors were more diverse (Supplementary Fig. 5h and Fig. 2d), with a small fraction of them in convergent orientation, and these loops appeared to be more distant (Supplementary Fig. 5i), suggesting these loops were likely to be related with domain formation. Many Hi-TrAC loop anchors did not have CTCF motifs (Fig. 2d and Supplementary Fig. 5h), suggesting different functions and organization mechanisms of these loops, and Hi-TrAC captured more versatile loops.

To investigate the relationship between chromatin loops and cell identity and activity, we further performed Hi-TrAC in another human cell-line, K562 cells (Supplementary Data 1). In total, 98,850 chromatin loops were identified in K562 cells (Supplementary Data 3). We hypothesized that cell-specific chromatin loops may control cell-specific gene expression. To test this, we compared the looping profiles in GM12878 and K562 cells, and identified 36,392 and 33,350 cell type specific loops, respectively (Supplementary Data 4). Cell-specific loops showed significant interaction signal enrichment in corresponding cells (Fig. 2e). We identified 4630 genes associated with GM12878-specific promoter loops and 7263 genes associated with K562-specific promoter loops. Meanwhile, we also identified 2251 genes that displayed alternative promoter-enhancer looping between GM12878 and K562 cells. Genes associated with GM12878-specific loops expressed at higher levels in GM12878 cells than in K562 cells; and vice versa (Fig. 2f). However, there were no significant differences in expression for the genes displaying alternative looping between GM12878 and K562 cells (Fig. 2f). We validated Hi-TrAC identified cell-specific loops with datasets from other methods through aggregation analysis, including in situ Hi-C, H3K27ac HiChIP and RAD21 ChIA-PET (Supplementary Fig. 6), demonstrating that Hi-TrAC successfully detected cell activity related differential chromatin loops.

The comprehensive regulatory interaction network was exemplified by several representative gene loci. EBF1 is a key transcription factor in B lymphopoiesis, which is expressed specifically in GM12878 cells, whereas GATA1 is a master transcription factor in erythropoiesis that is only expressed in K562 cells. *EBF1* and *GATA1* gene loci exhibited unique interaction patterns in corresponding cells, consistent with their expression profiles (Supplementary Fig. 7a, b). A previous elegant CRISPRi screening study identified multiple regulatory elements for *GATA1* expression^68^. Interestingly, taking the *GATA1* promoter as the viewpoint, the Hi-TrAC virtual 4 C signals correlated well with the CRISPRi score (Supplementary Fig. 7c, d), indicating the robustness of detecting functionally relevant regulatory interactions by Hi-TrAC.

Take another K562-specific key transcription factor gene *RUNX1* as an example (Supplementary Fig. 7e). Although the comprehensive regulatory network can be presented with interaction contact matrix heatmaps and loop arc plots, it is too complicated for visual inspection for each individual *cis*-regulatory element. Thus, we designed a Rehoboam plot to visualize chromatin loops of a specific genomic region, which clearly revealed individual loops connecting different cis-regulatory elements: the interactions were much stronger between the promoter and enhancers E6 and E20 in K562 than in GM12878 cells, which thus might be responsible for its expression in K562 cells (Fig. 2g).

We integrated all the enhancer and promoter loops, and generated a comprehensive regulatory interaction network (Fig. 2h). The degree of connection of enhancers and promoters in the network fitted the scale-free network power-law, revealing the complexity of the regulatory network (Fig. 2h and Supplementary Fig. 8a). The degrees of connection for enhancers were higher than those for promoters (Supplementary Fig. 8a), correlating with a high portion of enhancer-enhancer looping (Fig. 2c). On average, one enhancer directly interacted with one promoter, and one promoter had direct contacts with almost three enhancers, suggesting a redundant design of robustness for the cis-regulatory network (Supplementary Fig. 8b).

To test the functional contribution of direct and indirect enhancer loops to a target promoter in the interaction network, we chose the *CEMIP2* gene locus as a model, which has multiple potential enhancers (annotated as E1-E8) in K562 cells (Supplementary Fig. 8c). While E2 interacts directly with the promoter, both E3 and E5 interact strongly with E2, but not with the promoter. Interestingly, deleting any of these three potential enhancers by CRISPR/Case9 resulted in decreased expression of *CEMIP2* (Supplementary Fig. 8d). Meanwhile, the *CEMIP2* promoter interactions decreased by deleting any of these elements (Supplementary Fig. 8e). These results suggest that deleting one node of the interaction network may affect the stability of the whole regulatory interaction network, resulting in the dysregulation of gene expression.

### HCFC1 and ZNF143 are associated with promoter-centric chromatin looping

**Fig. 3:**
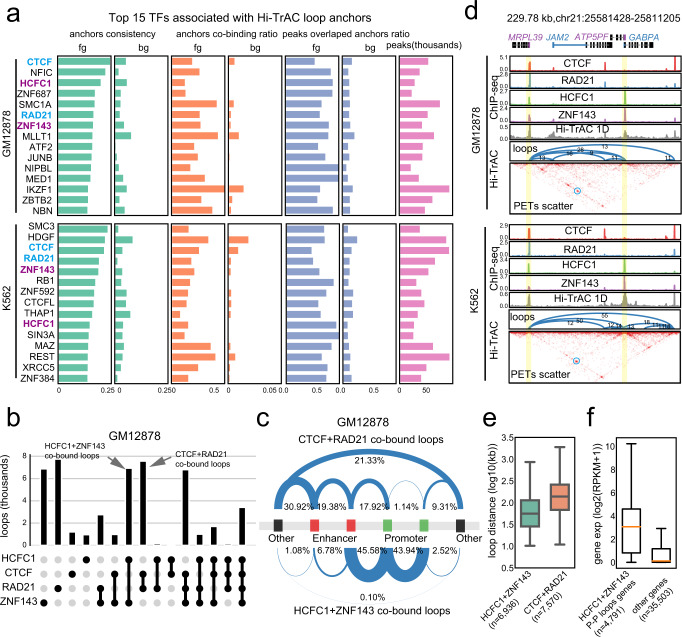
Division of labor in regulating different categories of chromatin loops by distinct transcription factors. **a** The top 15 transcription factors associated with the Hi-TrAC loop anchors in GM12878 and K562 cells. The binding sites of 162 and 360 transcription factors were compiled from the ReMap 2020^105^ for GM12878 and K562, respectively. The top 15 factors most significantly associated with loop anchors, sorted by consistency, are shown as indicated on the left of the panel (Supplementary Data 5**)**. Overlapped top factors between GM12878 and K562 are highlighted in purple and blue. Fg stands for foreground data, which means the actual loops. Bg stands for background data, which means regions nearby actual loop anchors and used as controls. Source data are provided as a Source Data file. **b** The distribution of loops (top panel) bound by HCFC1, CTCF, RAD21, and ZNF143 alone or in combination (bottom panel) at both anchors in GM12878. Source data are provided as a Source Data file. **c** Summary of loop categories with regard to putative cis-regulatory elements for loops co-bound by HCFC1 plus ZNF143 and loops co-bound by CTCF plus RAD21 in GM12878. **d** An example of promoter-promoter loops co-bound by HCFC1 plus ZNF143 but not CTCF plus RAD21. **e** The distribution of anchor distance for loops co-bound by HCFC1 plus ZNF143 and loops co-bound by CTCF plus RAD21 in GM12878. The box extends from the first quartile to the third quartile of the data, with a line at the median. The whiskers extend from the box by 1.5x the inter-quartile range. Flier points past the end of the whiskers were not shown. n = the number of loops. Source data are provided as a Source Data file. **f** The expression levels of genes with promoter-promoter loops co-bound by HCFC1 plus ZNF143 and other genes in GM12878. The box extends from the first quartile to the third quartile of the data, with a line at the median. The whiskers extend from the box by 1.5x the inter-quartile range. Flier points past the end of the whiskers were not shown. *n* = the number of genes. Source data are provided as a Source Data file.

We noticed that many loop anchors were not occupied by CTCF or SMC3 (Fig. 2a), especially for short-distance loops, suggesting that other factors may organize chromatin looping at these sites. To identify such potential factors, we analyzed the enrichment of 162 transcription factors (TFs) at Hi-TrAC loop anchors in GM12878 and 360 TFs in K562 cells (Supplementary Data 5). The top 15 enriched TFs in both cells included CTCF and RAD21 (Fig. 3a). Interestingly, the analysis also revealed that both cells shared two highly enriched TFs, HCFC1, and ZNF143, suggesting they may also be broadly involved in orchestrating chromatin looping. HCF1 and ZNF143 are ubiquitously expressed TFs that function at promoters of target genes, regulating cell metabolism, proliferation and differentiation^69–76^. Dysregulation of HCFC1 and ZNF143 is related with the pathogenesis of diseases (e.g. cancer)^77–80^. Accumulating evidence suggests that HCFC1 and ZNF143 may be involved in organizing chromatin structures^8,51–53,81–85^. The number of loops co-bound by HCFC1/ZNF143 but not CTCF or RAD21 was similar to the number of loops co-bound by CTCF/RAD21 but not HCFC1 or ZNF143 in GM12878 (Fig. 3b). Furthermore, our data indicated that only a small fraction of CTCF/RAD21 co-bound loops were associated with promoters, whereas a striking nearly 90% of promoter loops were anchored by HCFC1/ZNF143 binding (Fig. 3c, d). HCFC1/ZNF143 co-bound loops were generally shorter in genomic distance than CTCF/RAD21 loops (Fig. 3e). Genes with HCFC1/ZNF143 promoter-promoter loops showed higher expression levels than other genes (Fig. 3f). These results suggest a “division of labor” model for chromatin looping by different architectural proteins.

### Disrupting CTCF, RAD21, ZNF143, and HCFC1 results in distinct perturbation of looping

**Fig. 4:**
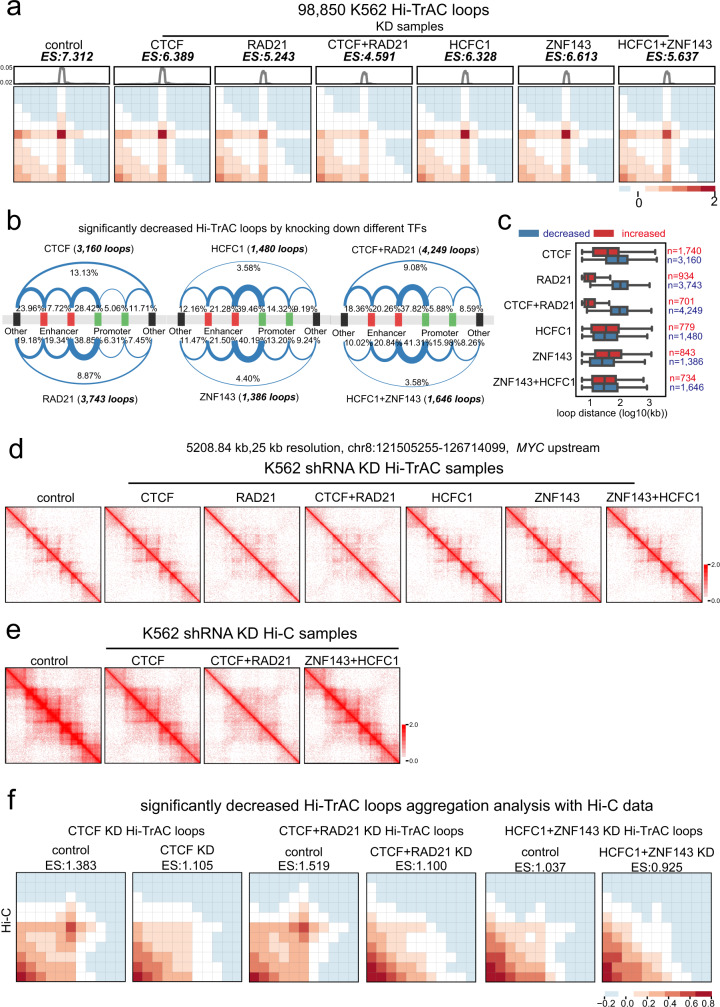
HCFC1 and ZNF143 contribute to organizing chromatin looping. **a** Aggregation analysis reveals decreases in chromatin looping intensity after knocking down CTCF, RAD21, HCFC1, and ZNF143 in K562 cells (Supplementary Data 6). Enrichment score (ES) is the mean value of all enrichment scores for individual loops. KD, knockdown. **b** Summary of loop categories with regard to putative cis-regulatory elements for decreased loops in the TF (transcription factor) knockdown cells. **c** The genomic distance distribution of changed loops after TF knockdown. The box extends from the first quartile to the third quartile of the data, with a line at the median. The whiskers extend from the box by 1.5x the inter-quartile range. Flier points past the end of the whiskers were not shown. n = the number of loops. Source data are provided as a Source Data file. **d** An example of domain disruption at the genomic region upstream of *MYC* gene detected by Hi-TrAC after knocking down RAD21 or CTCF with RAD21. **e** In situ Hi-C data shows the disruption of chromatin domains in the same region as in panel **d** after knocking down CTCF and RAD21 (Supplementary Data 8). **f** Loop aggregation analysis for significantly decreased loops detected by Hi-TrAC using the same in situ Hi-C data as in panel **e**.

To test the roles of CTCF, RAD21, HCFC1, and ZNF143 in maintaining chromatin looping, we knocked down (KD) these different factors either individually or in combination in K562 cells (Supplementary Fig. 9a, b). The resulting cells were then analyzed with Hi-TrAC (Supplementary Data 1). Aggregated enrichment scores of PETs in loop regions compared to nearby regions for all loops were used to compare the effect of knocking down on chromatin looping. While knocking down each of these TFs globally decreased the looping intensity, simultaneously knocking down both CTCF and RAD21 or both HCFC1 and ZNF143 resulted in more severe decreases in chromatin looping (Supplementary Data 6, Fig. 4a), suggesting that these TFs facilitate looping in general and they may act cooperatively to mediate looping. Consistent with this notion, each knocking down significantly decreased 3160 (CTCF KD), 3743 (RAD21 KD), 1480 (HCFC1 KD) and 1386 (ZNF143 KD) loops, respectively, while it enhanced smaller numbers of loops: 1740 (CTCF KD), 934 (RAD21 KD), 779 (HCFC1 KD) and 843 (ZNF143 KD), respectively (Supplementary Fig. 9c). Simultaneous knocking down of CTCF and RAD21 decreased 4249 and enhanced 701 loops, while simultaneous knocking down of HCFC1 and ZNF143 decreased 1646 and enhanced 734 loops, respectively (Supplementary Fig. 9c). While both decreased accessibility and decreased interactions were detected at a small fraction of accessible regions, the changes in chromatin looping intensity didn’t show a strong correlation with changes in accessibility at a global level (Supplementary Data 7, Supplementary Fig. 9d). As exemplified by two randomly picked regions (Supplementary Fig. 9e, f), among the 3160 decreased loops by knocking down CTCF, 412 loops showed no decreases in accessibility of anchors, suggesting that both chromatin interaction and accessibility may contribute to the Hi-TrAC signals. We found that 48.8% and 35.5% of the anchors of loops decreased by knocking down CTCF and RAD21, respectively, were other than enhancers and promoters, while lower fractions (24.93% and 25.11%, respectively) of the anchors of loops decreased by knocking down HCFC1 and ZNF143 belonged to this category of accessible regions (Fig. 4b). Similarly, the anchors of loops decreased by simultaneous knocking down of CTCF and RAD21 also displayed a higher fraction (36.03%) of non-promoter and non-enhancer regions than that (21.86%) decreased by the simultaneous knocking down of HCFC1 and ZNF143 (Fig. 4b). By comparison, knocking down of HCFC1 and ZNF143, either individually or simultaneously, resulted in higher fractions of disrupted enhancer- and promoter-related loops (Fig. 4b). The median sizes of loops decreased by knocking down CTCF and/or RAD21 were ~ 100 kb, while the median sizes of loops decreased by knocking down HCFC1 and/or ZNF143 were ~20–30 kb (Fig. 4c). These results indicate that HCFC1 and ZNF143 are involved in organizing different groups of chromatin loops compared with CTCF with RAD21.

To further validate the chromatin structure changes detected by Hi-TrAC, we performed in situ Hi-C with the control and knockdown cells (Supplementary Data 8). Consistent with what we observed in Hi-TrAC data (Fig. 4d), the in situ Hi-C data also showed severely compromised domain structures by knocking down CTCF and RAD21 but not by knocking down CTCF alone or simultaneously knocking down HCFC1 and ZNF143 (Fig. 4e). Hi-TrAC identified significantly decreased loops also showed decreases in the in situ Hi-C data of corresponding cells (Fig. 4f). These results demonstrate that Hi-TrAC can accurately detect alterations of chromatin structures.

To investigate the correlation between chromatin looping and gene expression, we analyzed the gene expression profiles in the control and knockdown cells (Supplementary Data 9). Knocking down each of these factors resulted in both up-regulated and down-regulated genes: 371 and 239 for CTCF, 926 and 925 for RAD21, 379 and 237 for HCFC1, and 255 and 466 for ZNF143, respectively (Supplementary Fig. 10a). Notably, the simultaneous knockdown of HCFC1 and ZNF143 down-regulated many more genes (646) than their individual knockdown. While there were generally no significant correlations between changes in gene expression and chromatin looping, a modestly positive Pearson Correlation Coefficient (PCC) of 0.219 for the down-regulated genes by simultaneously knocking down HCFC1 and ZNF143 was observed (Supplementary Fig. 10b), suggesting a possibility that the loops requiring both of these factors contribute to the expression of this group of genes. However, the data also indicated a complex relationship between changes in chromatin looping and gene expression, suggesting that HCFC1 and ZNF143-dependent loops could mediate either gene activation or repression.

### HCFC1 and ZNF143 work separately and synergistically with CTCF and RAD21

To determine if the changes in chromatin looping were direct consequences of depleting these TFs, we checked their chromatin binding profiles by ChIP-seq in knockdown cells (Supplementary Data 10). Simultaneously knocking down CTCF and RAD21 drastically reduced the binding of RAD21, whereas only reduced CTCF binding mildly (Supplementary Fig. 11a). Interestingly, the binding of HCFC1 was also impaired dramatically. Double knocking down of HCFC1 and ZNF143 significantly impaired their binding, and it also showed mild influence on the binding of CTCF and RAD21 (Supplementary Fig. 11a). HCFC1 and ZNF143 binding sites were enriched with the “ACTACANNTCCCA” ZNF143-associated motif (Supplementary Fig. 11b). Over 70% of HCFC1 and ZNF143 co-bound sites were located at promoters (Supplementary Fig. 11c). In both CTCF with RAD21 and HCFC1 with ZNF143 double knockdown cells, the top enriched motifs in decreased loop anchors included “CTCF” and “GATA” motifs (Supplementary Fig. 11d). Even though only 8% of the decreased loop anchors in HCFC1 and ZNF143 double knockdown cells were HCFC1 and ZNF143 co-bound peaks (Supplementary Fig. 11e), HCFC1/ZNF143 motif was still one of the top enriched motifs (Supplementary Fig. 11d). Furthermore, those decreased loop anchors not bound by HCFC1 or ZNF143 in the HCFC1 and ZNF143 double knockdown cells were enriched with “CTCF” and “GATA” motifs (Supplementary Fig. 11f). These binding motif analyzes of decreased loop anchors in knockdown cells suggest that HCFC1 and ZNF143 may act both separately and together with CTCF and RAD21.

### HCFC1 and ZNF143 orchestrate gene expression by organizing promoter loops

**Fig. 5:**
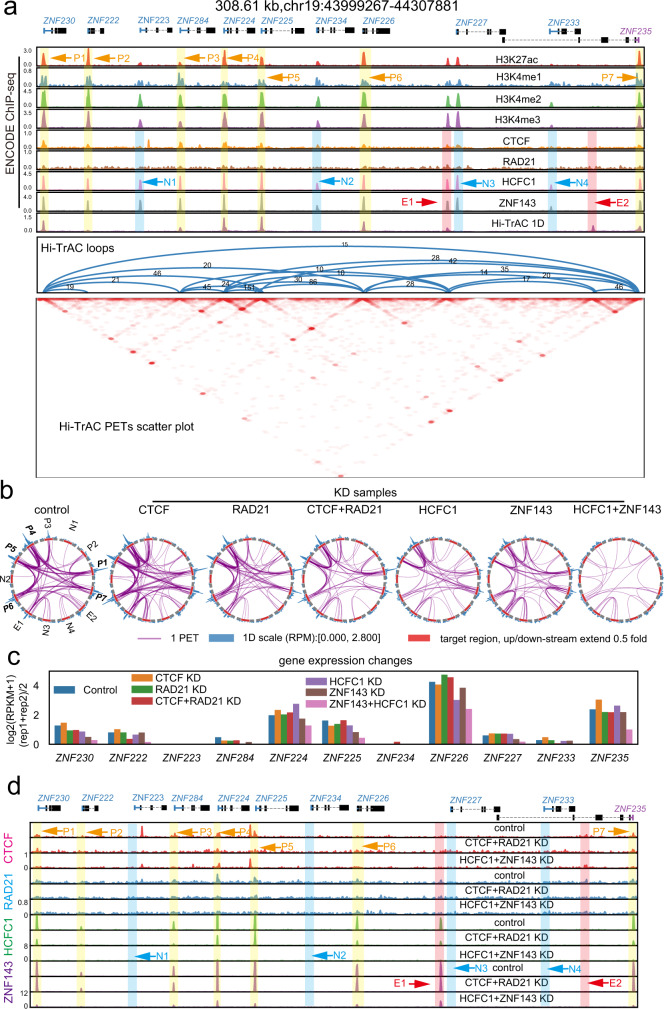
HCFC1 and ZNF143 regulate gene expression through organizing promoter-promoter looping. **a** Chromatin loops detected by Hi-TrAC are shown for the ZNF gene cluster on Chromosome 19 in K562 cells. Also shown are ENCODE ChIP-seq signals of active histone modifications and 4 TFs (H3K4me1, 2, 3, and H3K27ac, CTCF, RAD21, HCFC1, and ZNF143^115^). Putative promoters co-bound by HCFC1 and ZNF143 with loops are annotated as P1 to P7, and putative promoters co-bound by HCFC1 and ZNF143 but no loops detected are annotated as N1 to N4. The non-promoter region showing loops and co-bound by HCFC1 and ZNF143 is annotated as E1, and the region showing looping with other region but no HCFC1 and ZNF143 binding are annotated as E2. **b** Rehoboam plots of chromatin looping in control and knockdown cells for the ZNF cluster region as annotated in panel **a**. Source data are provided as a Source Data file. KD, knockdown. **c** The expression changes of the ZNF genes by knocking down CTCF, RAD21, HCFC1, and ZNF143 in K562 cells as measured by RNA-seq. **d** Binding profiles of CTCF, RAD21, HCFC1, and ZNF143 at ZNF gene cluster region detected by ChIP-seq in control, CTCF plus RAD21 double knockdown or HCFC1 plus ZNF143 double knockdown K562 cells.

To further investigate the functions of HCFC1 and ZNF143 in organizing chromatin structures, we examined a primate-specific genomic region that harbored multiple zinc-finger (ZNF) genes. This region exhibited strong promoter-promoter interactions, which is conserved between K562 and GM12878 cells. Multiple HCFC1 and ZNF143 binding peaks were detected at the loop anchors within this locus, whereas no strong CTCF and RAD21 binding was detected (Fig. 5a). The simultaneous knockdown of HCFC1 and ZNF143 severely compromised promoter-promoter looping within this region as shown by the Rehoboam plots (Fig. 5b), which was accompanied by decreased expression of the target genes (Fig. 5c), indicating a critical role of HCFC1 and ZNF143 in regulating looping and the expression of these genes. It was confirmed by ChIP-seq that the decrease in the promoter-promoter looping correlated with impaired bindings of HCFC1 and ZNF143 (Fig. 5d). We further validated the disruption of promoter-promoter looping detected by Hi-TrAC in the HCFC1 and ZNF143 knockdown cells using 3C-qPCR assays. The results indicated that both the *ZNF224*-*ZNF284* and *ZNF225*-*ZNF235* loops significantly decreased after knocking down HCFC1 and ZNF143 (Supplementary Fig. 12a). Two other randomly selected promoter-promoter loops outside of the ZNF gene cluster region, *MRPL24*-*PRCC* and *NDC1*-*TCEANC2*, were also significantly impaired by knocking down HCFC1 and ZNF143 (Supplementary Fig. 12a, b).

To further test whether the HCFC1 and ZNF143-dependent chromatin loops positively or negatively contribute to the expression of these genes, we deleted either ZNF225 or ZNF234 promoter loop anchor at the ZNF gene cluster region using CRISPR: the expressed *ZNF225* promoter is bound by HCFC1 and ZNF143 and interacts with other promoters in the region, while the non-expressed *ZNF234* promoter has only very weak binding of HCFC1 and ZNF143 and does not interact with other promoters and thus serves as a negative control (Fig. 5a–c). Deleting the *ZNF225* promoter loop anchor led to decreased interactions at *ZNF225* promoter and decreased *ZNF225* gene expression as expected (Supplementary Fig. 12c, d). Surprisingly, deleting *ZNF225* promoter anchor led to increases in loop formation at other promoters in this region, and also increases in the expression of those genes (Supplementary Fig. 12c, d). These results showed that HCFC1 and ZNF143 bound anchors contribute to promoter loop formation, which negatively affects the expression of nearby genes, potentially by directly competing for the limited transcriptional regulators.

## Discussion

We present here Hi-TrAC as a sensitive technique for mapping chromatin loops among transcription and chromatin regulator elements in accessible genomic regions. This technique shares the basic concept with Trac-looping, which utilizes Tn5’s ability to integrate DNA bridge into accessible chromatin regions to covalently link physically-contacting chromatin loci, thereby avoiding the proximity ligation that is required for the 3C-derived techniques^59^. Trac-looping needs 50–100 million cells and 5–7 days of bench work. Hi-TrAC is much more versatile and efficient, can be performed with as few as 0.01 million cells and less than two days of bench work. Moreover, it captures detailed interaction information with a resolution up to 200 bp with only 300 million sequencing reads, detecting interactions between regulatory elements with high sensitivity. Chromatin interactions detected by Hi-TrAC are highly correlated with those detected by Micro-C, MCC and even CRISPRi, suggesting that Hi-TrAC can reliably capture spatial contacts of regulatory elements. Moreover, comparing to Micro-C, Hi-TrAC detected four times more chromatin loops (300k vs 64k) with only 10% of PETs (223 M vs 2396 M); Hi-TrAC detected almost all the interactions between potential enhancers and their target promoters as detected by MCC. Comparing to other 3C-based techniques, Hi-TrAC also showed advantage in detecting much more chromatin loops at high resolution with less sequencing amount. Thus, Hi-TrAC can serve as an inexpensive, highly sensitive and robust alternative method for analyzing interactions among regulatory elements of chromatin and transcription.

The bridging linker used in Hi-TrAC works like a ruler charting the genome map. To be bridged by the linker, the spatial distance between two interacting loci should be shorter than the length of the linker. We tested linkers with different lengths and found that a linker with a 30 bp spacer between the flanking Tn5 binding sites performed best. Longer linkers captured too many inter-chromatin interactions, whereas shorter linkers lost many distal interactions (Supplementary Data 11). The chosen bridging linker is estimated as ~20 nm long, suggesting the spatial physical distance between Hi-TrAC captured interacting regions are within that range.

Enhancer-promoter interaction loops play a critical role in controlling the temporospatial expression of genes^31,33,35,36,38,40,43^. Taking the β-globin locus as an example, the expression switching of fetal and adult hemoglobin is regulated by interactions between a locus control region (LCR) and promoters of corresponding genes with the help of transcription factors^86,87^. To gain comprehensive information on the mechanisms of gene regulation genome-widely, it is important to identify the regulatory interaction networks of enhancers and promoters. However, even the most comprehensive 3D data in previous studies only provide limited information on genome-wide enhancer-promoter loops^8,61^. To our knowledge, we now have provided the most comprehensive interaction data among accessible chromatin regions in GM12878, K562 cells, and mESCs, reporting a total of about 500,000 chromatin loops. Further, our data also revealed shared chromatin loops and cell-specific loops that contribute to cell-specific expression of genes that are responsible for differentiation and cellular function. Even for genes showing similar expression levels in both cell types, the interactions of regulatory elements, especially of enhancers, exhibit different patterns, suggesting that gene expression could be differentially regulated in different cells.

Chromatin loops can be roughly separated into three categories based on the location of loop anchors relative to chromatin domain organization: (1) in the TAD boundaries; (2) in the sub-TAD boundaries; and (3) within TADs and sub-TADs. They can also be categorized based on the functional annotation of the loop anchors: (1) promoters; (2) enhancers; and (3) others. While the loops linking enhancers and promoters are often found within TADs or sub-TADs, others are mapped to the boundaries of these chromatin domains. Generally, the loops originating from the domain boundaries are much longer (>100 kb) than those linking enhancers and promoters (<100 kb). Previous data have established that CTCF and cohesin complex play critical roles in maintaining the chromatin interaction of TADs^48,50,88–90^ via an extrusion model^18–23^. Several specific transcription factors have been found to contribute to chromatin looping between enhancers and promoter^51–58^. YY1 facilitates the loop formation between enhancer and promoter and regulates gene expression, and ZNF143 was reported to act with CTCF to facilitate chromatin looping. However, in general, the mechanisms that regulate enhancer-promoter looping require much clarification. In this study, we found that in addition to CTCF and RAD21, ZNF143 and HCFC1 are also among the top enriched factors and shared among GM12878 and K562 cells, suggesting that these two factors may play a general role in chromatin looping. Interestingly, over 96% of the loop anchors co-bound by ZNF143 and HCFC1 in GM12878 cells involved enhancers and promoters, while only 38% of the loop anchors were co-bound by CTCF and RAD21 in GM12878 cells involved enhancers and promoters, suggesting that ZNF143 and HCFC1 play a general role in contributing to enhancer-promoter loops. The loops disrupted by knocking down HCFC1 and/or ZNF143 showed a median size of 20 kb, consistent with the size of enhancer and promoter interactions, while the loops disrupted by knocking down CTCF and/or RAD21 showed a median size of about 100 kb, consistent with the distance between domain boundaries.

Although it was previously suggested that promoter-promoter interactions may facilitate gene expression by bringing the target promoters in close proximity^29,91–93^, direct data to support the hypothesis are needed. Here, we found that HCFC1 and ZNF143 bound to the anchors of promoter loops at the ZNF gene cluster locus; the simultaneous knocking down HCFC1 and ZNF143 disrupted promoter-promoter loops and decreased the expression of these target promoters and thus providing strong evidence that the promoter-promoter looping contributes to the gene expression. However, since chromatin accessibility is necessary for Hi-TrAC to detect chromatin interactions, the decreased interactions detected by Hi-TrAC could be derived from either decreased interaction alone or from decreased accessibility and interactions together, both of which could be regulated by HCFC1 and ZNF143. Based on these results, we propose that different architectural proteins show a division of labor for organizing chromatin looping: CTCF and RAD21 are responsible for building the outer frame of chromatin domains, whereas HCFC1 and ZNF143 decorate the inner structures by organizing looping between regulatory elements. With the development and completion of high-resolution genome structure and more chromatin binding datasets of transcription factors, more chromatin looping regulators will be identified, including general ones and cell type-specific ones. Our comprehensive data on the interaction network among accessible chromatin regions provide a rich resource to further explore the complex function and mechanisms in genome organization.

## Methods

### Cell-lines

GM12878 cells were purchased from Coriell Institute; K562 (CCL-243), 293 T (CRL-3216), mES-E14TG2a (CRL-1821), and mES-R1 (SCRC-1011) cells were purchased from ATCC.

### Hi-TrAC experimental procedures

Cells were fixed with 1% Formaldehyde in a culture medium at room temperature for 10 min. Wash the cells twice with 1 mL ice-cold PBS, then keep cells on ice. The Tnp complex was assembled by mixing 2 μL short adapter (50 μM), 2 μL bridging linker (25 μM) (Supplementary Data 11), 2 μL glycerol and 4 μL Tn5 (100 μM), then incubated at room temperature for 20 min. Resuspend cells with 100 μL reaction buffer (50 mM Tris-acetate, pH 7.5, 150 mM potassium acetate, 10 mM magnesium acetate, 4 mM spermidine, 0.5% NP-40), and incubate at room temperature for 10 min. Add 10 μL Tnp complex to the permeabilized cells, then mix gently by pipetting and incubate on a 37 °C thermomixer for 4 h with interval mixing. The reaction was stopped by adding EDTA (25 mM final concentration) and SDS (0.3% final concentration). Add 2 μL protease K (20 mg/mL) to the reaction mixture and incubate at 65 °C overnight to reverse crosslinking. DNA was purified by Phenol-Chloroform extraction. The gaps in DNA were repaired by T4 DNA polymerase in the reaction mixture containing dNTPs at room temperature for 30 min. The free bridging linker (68 bp) was removed by selectively binding large DNA fragments (>100 bp) to AMPure XP beads. The DNA was eluted from AMPure XP beads with 80 μL elution buffer and digested with the restriction enzymes 2 μL MluCI (NEB, R0538L) and 2 μL NlaIII (NEB, R0125L) in 100 μL reaction mixture at 37 °C for 30 min. The reaction mixture was adjusted to 1x B&W buffer by adding 100 μL 2x B&W buffer (10 mM Tris-HCl, pH 7.5, 1 mM EDTA, 2 M NaCl, 0.1% Tween-20) and then mixed with 5 μL Streptavidin C1 beads (Invitrogen, 65001) for 30 min with rotation. The beads were washed 5 times with 1 mL 1x B&W buffer. Biotin-labeled DNA fragments captured on beads were ligated to multiplexing adapters by adding 5 μL each adapter (50 μM) and 1 μL T7 DNA ligase (NEB, M0318L) in 100 μL ligation mixture and incubating at room temperature for 1 h with rotation. Before PCR amplification, the beads were washed 5 times with 1x B&W buffer. The Hi-TrAC libraries were then amplified with multiplexing indexed primers in the following reaction mixture: 20 μL Phusion HF PCR Master Mix (NEB, M0531S), 1 μL Illumina Multiplexing PCR primer 1.0 (10 μM), 1 μL Illumina Multiplexing PCR index primer (10 μM) and 18 μL H_2_O for 12 cycles. The DNA fragments between 300 bp and 700 bp were excised for paired-end sequencing on Illumina platforms.

### Knockdown and western blotting

Knockdown of CTCF, RAD21, HCFC1, and ZNF143 in K562 cells was performed by transduction with shRNA lentivirus. shRNA templates were cloned into pGreenPuro lentivector (System Biosciences, SI505A-1). shRNA targets are: Control-shRNA-1: GCGCGATAGCGCTAATAATTT, Control-shRNA-2: CAACAAGATGAAGAGCACCAA; CTCF-shRNA-1: GGAGAAACGAAGAAGAGTA, CTCF-shRNA-2: GTAGAAGTCAGCAAATTAA; RAD21-shRNA-1: AGAGTTGGATAGCAAGACA, RAD21-shRNA-2: GGAAGCTAATTGTTGACAGTGTCAA; HCFC1-shRNA-1: GCAACCACCATCGGAAATAAA, HCFC1-shRNA-2: AGAACAACATTCCAAGGTACCTGAA; ZNF143-shRNA-1: GCTACAAGAGTAACTGCTAAA, ZNF143-shRNA-2: GGACGACGTTGTTTCTACACAAGTA. Co-transfect 12 μg lentivector with packaging plasmids 9 μg psPAX2 (Addgene, #12260) and 3 μg pMD2.G (Addgene, #12259) into 293 T cells cultured in 100 mm dish. Change with 12 mL fresh medium at 12 h after transfection, and medium supernatant containing virus was collected at 72 h after transfection. Add the medium to 3 million K562 cells to start transduction. Cells were harvested 72 h after infection. Protein expression was checked by western blotting. We used Invitrogen NuPAGE gel electrophoresis system following the owner’s manual, and proteins were transferred onto PVDF membrane. Primary antibodies used for detecting corresponding proteins are: anti-CTCF (Cell Signaling Technology, 3418 S, dilution 1:1000), anti-RAD21 (Abcam, ab217678, dilution 1:1000), anti-HCFC1 (Santa Cruz Biotechnology, sc-390950, dilution 1:1000) and anti-ZNF143 (Abnova Corporation, H00007702-M01, dilution 1:1000).

### RNA-seq library construction

Total RNA from 5000 cells was extracted and purified with QIAzol Lysis Reagent (QIAGEN) and RNeasy mini kit (QIAGEN). RNA-seq library was constructed with purified RNA following the Smart-seq2 protocol^94^.

### Generating loop anchor deletion cells

CRISPR targeting sequences were designed and cloned into pSpCas9(BB)−2A-Puro vector (Addgene #62988). Following transfection of K562 cells with Cas9 and sgRNA expressing plasmids for 24 h, the cells were treated with 2 μg/mL Puromycin for 48 h to kill non-transfected cells. Surviving cells were sorted into 96-well plate at a density of one cell per well, cultured for two to three weeks, and genotyped using specific PCR primers for identification of loop anchor deletion clones. The targeting sequences used are following: CEMIP2-E2, 1-GATCGAGTTCTAGTTGACCC, 2-GTGCGTCTATGAATCTGCGC; CEMIP2-E3, 1-GTAAGCACATGGCCCGTCAG, 2-TCGAACAGGAACGTACTATC; CEMIP2-E5, 1-CTAACGCAATCCACCTAGAA, 2-TAAGGCTCTCTACTTAGCGG; ZNF225-promoter, 1-TGGCGCTTAACGACGAACCC, 2-TTTATGGGGCACGGCGACCA; ZNF234-promoter, 1-AAGGAGGATCCTATACGTGA, 2-TAAGCCGCAACGTGACTCTG.

### Public data and pre-processing

Public data used in this study, including Hi-C, HiChIP, ChIA-PET, capture Hi-C, MCC, Micro-C, RNA-seq, ATAC-seq, DNase-seq, and ChIP-seq, were summarized in Supplementary Information. Biological and technical replicates were merged for the same factor, and only unique reads were used for the following analyses.

### Public genomic annotations

Human (gencode.v30.basic.annotation.gtf) and mouse (gencode.vM21.basic.annotation.gtf) gene annotations from GENCODE^95^ were used in any gene-related analysis. Human genome version hg38 and mouse genome version mm10 were used in this study. If human or mouse data are generated in other genome versions, they are always converted to hg38 or mm10 for analysis.

Putative enhancer and promoter annotations of human cells were obtained from NIH Roadmap Epigenomics Consortium^96^ and processed as following: 1) all regions annotated as “Enh” and “Tss” were collected; 2) overlapping regions were merged with BEDtools merge; 3) merged regions were further annotated by annotatePeaks.pl in HOMER package^97^ with gene annotation file downloaded from GENCODE^95^; if a region is within 2 kb either upstream or downstream of a TSS, it is defined as a promoter; otherwise it is defined as an enhancer. 4) neighboring enhancers or promoters with gaps <100 bp were merged again by BEDtools merge.

Putative cis-regulatory elements of mouse embryonic stem cells were defined by ATAC-seq peaks. If a peak is within 2 kb either upstream or downstream of a TSS, it is defined as a promoter; otherwise, it is defined as an enhancer.

### Pre-processing of Hi-TrAC data

Raw paired-end reads in FASTQ files were first trimmed of the linker sequence CTGTCTCTTATACACATCT from both ends. Only paired-end tags (PETs) with both ends with a length ≥ 10 bp were kept. Trimmed PETs were mapped to hg38 using Bowtie2^98^ with –end-to-end–very-sensitive parameter. Mapped PETs with MAPQ ≥ 10 were converted to BEDPE files. Mapped PETs with a distance shorter than 1 kb without linker sequence in any end were further filtered. PCR replicates of PETs were filtered if the locations of both ends were identical. Unique intra-chromosomal PETs (cis PETs) as BEDPE files were mainly used for downstream analysis. All the described processing steps were summarized as tracPre2.py in the cLoops2 package^99^. Quality control statistical results were also generated by tracPre2.py. BEDPE files were used to analyze PET level properties, and the cLoops2 pre-module processed them to cLoops2 data directories for other analyses such as domain-calling, loop-calling, and visualization.

### Virtual 4C signals of Hi-TrAC or Micro-C

Virtual 4 C signals were generated by only keeping the PETs with one end located within 1 kb either upstream or downstream of target TSSs. These PETs were then piled up as the 1D signal. The method is implemented in the cLoops2 plot module for visualization or the cLoops2 dump module for data extraction^99^.

### Comparisons between mESC Hi-TrAC and Micro-C loops

Micro-C loops were called by HiCCUPS in Juicer package (v1.22.01)^100^ with parameter settings of –cpu –ignore-sparsity -r 2500 -f 0.1 -k KR -p 4 -i 8 -d 2 for the 2.6 billion PETs HIC file downloaded from GEO, according to the original paper as leading to the most of loops compared to other resolutions^60^. More loops were called from the Micro-C data than the original paper due to the upgrades of the HiCCUPS algorithm^60^. The loop-calling algorithm described in cLoops^101^ was slightly improved and implemented as the cLoops2 callLoops module^99^ for loop-calling with Hi-TrAC data. mESC Hi-TrAC loops were called by the cLoops2 callLoops module with parameters of -eps 200,500,1000,2000 -minPts 20 -p 30 -w -j -i -max_cut -cut 5000. For the overlapping analysis, loop anchors were extended to 5 kb, unique and overlapped loops were obtained by pairtopair subcommand in BEDTools (v2.29.2) package^102^ with -type notboth or -type both options.

### Calling loops from Hi-TrAC data of human cells

The cLoops2 callLoops module with key parameters settings of -eps 200,500,1000,2000 -minPts 10 -max_cut was used to call loops from GM12878 Hi-TrAC data, requiring a loop supported by at least 10 PETs. For K562 Hi-TrAC data, loops were called by parameters settings of -eps 200,500,1000,2000 -minPts 10 -cut 5000 to filter PETs with distance short then 5 kb, by which the default parameters will automatically filter all loops short than 20 kb.

### Loop aggregation analysis

An 11 × 11 contact matrix was constructed for a loop from interacting PETs, together with its five upstream and downstream windows of the same size. An individual enrichment score for a loop was calculated as the number of PETs in the 11 × 11 contact matrix center divided by the mean value of all others. The global enrichment score was the mean value of all enrichment scores for individual loops. The 11 × 11 contact matrix was further normalized by the total number of PETs in the matrix and z-score normalization. Heatmap was plotted of the average matrix for all normalized 11 × 11 matrices. The analysis was implemented in cLoops2 agg module with the option of -loops. Except for the parameters specifically mentioned, the default parameter with -loop_norm was used to generate visualization results.

### CTCF motif orientations

The whole-genome-wide CTCT motif orientations were annotated by FIMO^103^ with CTCF motif recorded in CIS-BP database^104^.

### Calling differential loops

Loops from samples under different conditions were combined and quantified in both conditions. The neighboring regions nearby loop anchors, which were the permutated nearby background regions defined in cLoops for estimation loops statistical test, were also quantified. The background data for the two conditions were fitted linearly. The fitted linear model was used to transform the PETs in loops of the treatment dataset to the control set, assuming there should be no difference in background data. False discovery rate (FDR) is a required parameter to find the cutoffs of average and fold change in the background data MA plot. The cutoffs were then applied to the transformed loops data. Poisson p-values were finally assigned to each loop as follows,

1 $$p=1-\mathop{\sum }\limits_{i=1}^{{fg}-1}{Poisson}(i,\,{{\max }}({bg},\,{fgNearby},\,{bgNearby},\,{pseudo}))$$

Where fg stands for the bigger value of PETs in the testing loop for treatment vs. control, bg stands for the smaller value for comparison, fgNearby is the number of PETs for background data for the testing condition, and bgNearby is the number of PETs for background data for the control condition. Pseudo is a general noise control value, set to 1 for all times. All numbers except pseudo here are transformed by the linear fitting above the background data. The P-values were corrected by Bonferroni correction, and by default 0.01 was used as the cutoff for significance.

This algorithm was implemented in the cLoops2 callDiffLoops module^99^, and differentially enriched loops were called with key parameters of -fdr 0.05 for GM12878 vs. K562 Hi-TrAC data.

### Transcription factors associated with Hi-TrAC loops

Integration of public ChIP-seq data and Hi-TrAC loops was intended to identify transcription factors associated with chromatin looping. We collected the binding sites for 162 transcription factors in GM12878 and 360 transcription factors in K562 from ReMap 2020^105^ (remap2020_all_macs2_hg38_v1_0.bed) on 2020-08-09. For a loop, both the regions of three sizes upstream of its left anchor (smaller coordinate in the genome) and downstream of its right anchor (bigger coordinate in the genome) were linked as background data (false loops) for comparisons, to calculate the enrichment of TF binding sites on both anchors. Any background regions overlapping with true loop anchors were removed. The overlaps between anchors and background regions with transcription factor binding sites were compiled into the left anchors’ matrix and the right anchors’ matrix. Rows are anchors or backgrounds, and columns are factors in the binary matrix. In the binary matrix, 1 indicates the anchor is bound by the factor and 0 stands for no binding. With the two binary matrices, the following attributes were calculated and used to find enriched transcription factors: 1) anchors consistency: for a TF, a vector from the left anchor matrix and a vector from the right anchor matrix were used to calculate the Spearman correlation coefficient, indicating the co-binding consistency of a factor at both anchors. 2) anchors co-binding ratio: for a TF, the ratio of anchors bound by the TF. 3) TF peaks overlap ratio: for a TF, ratio of peaks overlapping with loop anchor regions. To filter TFs, the following cutoffs were used: 1) comparing to the background, ratio of consistency >2; 2) anchors co-binding ratio >0.1; 3) comparing to the background, the ratio of co-binding ratio >2; 4) comparing to the background, the ratio of TF peaks overlap ratio ≥ 1. Except for using the anchor flanking regions as background, we also implemented the random shuffling value 1000 times as background to ensure the observed attributes are higher than permutation background and require FDR < 0.001. The remaining TFs were sorted by consistency in descending order, by which known looping associated factors such as CTCF and cohesin are among the top-ranked factors.

### Analysis of the Hi-TrAC data from the control and TF knockdown K562 cells

Raw Hi-TrAC data were processed by tracPre2.py first to extract the unique cis PETs (Supplementary Data 1). Aggregated loops analysis was performed to obtain the enrichment scores of all loops called from K562 Hi-TrAC data and to check the consistency among two shRNAs and biological replicates. All unique cis PETs from the same TF knockdowns were pooled together and sub-sampled to 74 million (the pooled control sample has the least PETs of 74.37 million) for all downstream analyses. The global enrichment scores were also used to show the global changes in all or subsets of K562 Hi-TrAC loops in the TF knockdown samples. Differentially enriched loops from the knockdown samples to control samples (all loops called from K562 Hi-TrAC data were used as the comparing set) were called with key parameters of -noPCorr -pcut 0.001, by which the above-uncorrected Poisson test *P*-value 0.001 was used to select significantly changed loops.

### Rehoboam plots for visualization of interaction changes

In a Rehoboam plot, each putative cis-regulatory element inferred from Hi-TrAC loops or other sources with extended nearby regions was shown as a part of a circle, Hi-TrAC 1D profiles were shown outside the circle, and Hi-TrAC interaction densities were shown as the widths of arches or each Hi-TrAC PET shown as an arch among circle parts. Viewpoints can be set only to keep the PETs oriented from some of the elements. We name the visualization result as a Rehoboam plot because it looks like a predicted divergence from the AI system named Rehoboam in WESTWORLD Season Three. This visualization method is implemented in the cLoops2 montage module^99^.

### Analysis of Hi-C data from the control and TF knockdown K562 cells

Raw Hi-C data reads were processed to human reference genome hg38 by HiC-Pro (v2.11.1)^106^. Only intra-chromosomal PETs from the files with a suffix of allValidPairs output by HiC-Pro were used for all following analyses. The cLoops2 plot module was used to generate visualization plots. Replicates of the Hi-C libraries were combined to validate the decreased loops detected by Hi-TrAC with aggregation analysis, and only the loop and nearby region with more than 20 PETs were used to do the analysis considering the sparsity of Hi-C interacting PETs.

### Analysis of RNA-seq data from the control and TF knockdown K562 cells

Raw RNA-seq data reads were mapped to human reference genome hg38 by STAR (v2.7.3a)^107^. Gene annotation file (v30) downloaded from GENCODE^95^ was used to quantify gene expression level by Cufflinks (v2.2.1)^108^. Further, significant differentially expressed genes (knocking down samples vs. control) were called by Cuffdiff (v2.2.1) in Cufflinks package, requiring *P*-value <0.001 and fold changes ≥1.

### ChIP-seq

Control and TF knockdown K562 cells were fixed with 1% Formaldehyde in culture medium at room temperature for 10 min. Wash the cells twice with 1 mL ice-cold PBS, then keep cells on ice. 0.1 million fixed cells were used for CTCF and RAD21 ChIP-seq library preparation, and 0.5 million cells were used for HCFC1 and ZNF143 ChIP-seq library preparation. Resuspend cells with 1x TE buffer provided with 1 mM PMSF and 1x protease inhibitor cocktail. Chromatin shearing was performed on a Diagenode Bioruptor Pico sonication device at 4 °C for 6 cycles with 30 sec on and 30 sec off, resulting in 200–1000 bp fragments. Adjust chromatin solution to 1x RIPA buffer (1x TE, 0.1% SDS, 0.1% Sodium Deoxycholate and 1% Triton X-100) plus 200 mM NaCl. Collect the chromatin supernatant after centrifugation at 15,000 × *g* for 10 min in a 4 °C microcentrifuge. 10% of the chromatin supernatant was saved as input. Mix 2 μg antibody with 20 μL Dynabeads Protein A beads (ThermoFisher Scientific, Cat. 10001D) with rotation at room temperature for 1 h. Wash the beads once with 1× PBS, then add chromatin solution to beads, and incubate at 4 °C overnight with rotation. Wash the beads twice with RIPA buffer, then twice with RIPA buffer plus 300 mM NaCl, then twice with LiCl buffer (1x TE, 250 mM LiCl, 0.5% NP-40, 0.5% Sodium Deoxycholate), and finally twice with 1x TE buffer. Elute DNA and reverse crosslinking by protease K digestion and incubating at 65 °C for 6 h. Purify DNA with MinElute Reaction Cleanup Kit (QIAGEN, Cat. 28206). Repair ends of DNA using End-It DNA End-Repair Kit (Lucigen, Cat. ER0720), then perform A-tailing with Klenow Fragment (3’-> 5’ exo-) (NEB, Cat. M0212S) provided with dATP, and then perform adapter ligation with T4 DNA ligase (NEB, Cat. M0202L). Amplify the library and add index by PCR. DNA fragments between 200 bp and 600 bp were purified and sequenced on Illumina platforms.

The following antibodies were used in the experiments: anti-CTCF (Cell Signaling Technology, Cat. 3418 S), anti-RAD21 (Abcam, Cat. ab217678), anti-HCFC1 (Cell Signaling Technology, Cat. 69690 S) anti-ZNF143 (Abnova, Cat. H00007702-M01).

### ATAC-seq

ATAC-seq was performed with 50,000 cells following the protocol as reported^109^.

### 3C-qPCR

Resuspend 1 million fixed cells with 1 mL ice-cold lysis buffer (10 mM Tris-HCl, pH 8.0, 10 mM NaCl, 0.2% NP-40, 1 × protease inhibitor), then incubate on ice for 20 min. Collect cells by centrifugation at 4 °C, then resuspend with diluted CutSmart buffer (346 μL H_2_O, 50 μL 10× CutSmart Buffer, 44 μL 1% SDS). Incubate at 65 °C for 10 min. Add 50 μL of 10% Triton X-100, then shake on a thermomixer at 37 °C for 1 h. Add 100 U selected restriction enzyme (for *MYC*_promoter-enhancer, use SpeI; for *ZNF224*-*ZNF284*, use BamHI; for *ZNF225*-*ZNF235*, use HindIII; for *MRPL24*-*PRCC* and *NDC1*-*TCEANC2*, use NcoI), then incubate at 37 °C overnight with shaking. Collect digested nuclei by centrifugation, then resuspend with 100 μL inactivation buffer (1 × PBS, 1% SDS), and incubate at 65 °C for 20 min. Add 895 μL diluted T4 ligation buffer (695 μL H_2_O, 100 μL 10 × T4 DNA Ligase Reaction buffer, 100 μL 10% Triton X-100) and mix well. Add 100 U T4 DNA ligase, and incubate at 16 °C overnight. Add 30 μL 10% SDS and 100 μg Protease K to stop the ligation reaction, then incubate at 65 °C to reverse crosslinking. Purify 3 C libraries by Phenol-Chloroform extraction. Design primers and probes according to the sequence of the interaction pair, then quantify the interaction frequency by qPCR. The promoter region of *MYC* gene was used as input control. Used primers and probes are: *MYC* promoter input, forward: CTCAGCAGCAGCTCCAAATA, probe: /56-FAM/AGAGTGCTG/ZEN/CTAGAGCAACAAGCA/3IABkFQ/, reverse: GACCATGGAAGTTGCCTTCT; *MYC* promoter-enhancer, forward: TCATTTCAGGGAGCAAACAAATC, probe: /56-FAM/ACGCTTCGA/ZEN/CTTAGCTAGTTGCCC/3IABkFQ/, reverser: TTACTCTGGAATAGGTTCCATGC; *ZNF224*-*ZNF284*, forward: GACTGGTGGTCTCTTCTTAGTG, probe: /56-FAM/ATTTCCCAC/ZEN/GAAGCCTGTCAGGTC/3IABkFQ/, reverse: TCGATCACCAGTTCTTTGAGG; *ZNF225*-*ZNF235*, forward: GTAGCTGGATCTCCTAGACTCA, probe: /56-FAM/AGGAGTTTC/ZEN/CAAACAACAGGCGTCT/3IABkFQ/, reverse: ATTAACATTGTATCAAATATTGCTCAACCA; *MRPL24*-*PRCC*, forward: GCCTGGCACATACTGAATACT, probe: /56-FAM/AAAGGATAG/ZEN/GCTCTTCCCGCACC/3IABkFQ/, reverse: GCGGAAAGTGGAGGTGAG; *NDC1*-*TCEANC2*, forward: AGACCGAGTCAAATGCTTCAG, probe: /56-FAM/TAGTCTAGG/ZEN/GCGTACAGGAGACCG/3IABkFQ/, reverse: GCCTTCCTGCCTTTGAACT.

### Analysis of ChIP-seq and ATAC-seq data from the control and TF knockdown K562 cells

Raw ChIP-seq and ATAC-seq data reads were mapped to human reference genome hg38 by Bowtie2^98^ with key parameters of –local –very-sensitive –no-unal –no-mixed –no-discordant. Mapped PETs with MAPQ > =10 were converted to normalized signals (reads per million) as bigWig files by deepTools^110^ for visualization or aggregation analysis.

### Motif analysis

Motif analysis for Hi-TrAC loop anchors or ChIP-seq peaks was performed by findMotifsGenome.pl in HOMER package^97^. Only top-ranked significant known motifs were shown.

### GO terms enrichment analysis

GO terms enrichment analysis for genes was performed by script findGO.pl in HOMER package^97^, requiring more than ten overlapping genes in the terms, and there are fewer than 1000 genes in the terms. Only top-enriched terms sorted by *P*-values were shown.

### Data visualization

Most of 1D profile and heatmap visualizations were shown by the cLoops2 plot module. Networks were visualized and analyzed by NetworkX^111^. Other plots were generated by matplotlib^112^ and seaborn^113^.

### Statistics & reproducibility

No statistical method was used to predetermine sample size of Hi-TrAC libraries. No data were excluded from the analyses. The experiments were not randomized.

### Reporting summary

Further information on research design is available in the Nature Research Reporting Summary linked to this article.

## Supplementary information

Supplementary Information

Description of Additional Supplementary Files

Supplementary Data 1

Supplementary Data 2

Supplementary Data 3

Supplementary Data 4

Supplementary Data 5

Supplementary Data 6

Supplementary Data 7

Supplementary Data 8

Supplementary Data 9

Supplementary Data 10

Supplementary Data 11

Reporting Summary

## Source data

Source Data

## Data Availability

Hi-TrAC, RNA-seq, ATAC-seq, Hi-C, and ChIP-seq data generated by this study have been deposited to GEO under accession code GSE180175 and to SRA under accession code SRP328503. Source data are provided with this paper.

## Code availability

Major analyses of loop-calling and cell or condition specific loop-calling are summarized in the cLoops2 package and freely available at GitHub with documentation, test data and updates: https://github.com/YaqiangCao/cLoops2. The package is also available at Zendo: https://zenodo.org/record/7140320#.YzsjWy-B0UE.
