# Supplementary material for: Hi-TrAC reveals division of labor of transcription factors in organizing chromatin loops
Source: Nat Commun. 2022 Nov 5;13:6679. doi: 10.1038/s41467-022-34276-8 (PMC9637178; doi:10.1038/s41467-022-34276-8)
Supplement: Supplementary file 1 — Supplementary Information [file 41467_2022_34276_MOESM1_ESM.pdf]

Supplementary Information for

Hi-TrAC reveals division of labor of transcription factors in organizing chromatin loops

Shuai Liu[1,2], Yaqiang Cao[1,2], Kairong Cui[1], Qingsong Tang[1], and Keji Zhao[1,3]

1. Laboratory of Epigenome Biology, Systems Biology Center, Division of Intramural Research, National Heart, Lung and Blood Institute, National Institutes of Health, Bethesda, Maryland, USA
2. These authors contributed equally to this work.
3. Corresponding author

Supplementary Methods

Public data

Used public datasets were summarized in **Supplementary Table 1**.

Supplementary Table 1: Public datasets used in this study.

Accession	Data Type	Cell Type	Factor	Reference
GSM1551619 (GEO)	Hi-C	K562	None	[1]
ENCLB620YVM, ENCLB779ZWL, (ENCODE)	ChIA-PET	K562	RAD21	[2]
GSM2705045, GSM2705044, GSM2705043, (GEO)	HiChIP	K562	H3K27ac	[3]
GSM1155963, GSM1155962, GSM1155961, GSM1155960, GSM1155959, GSM1155958, GSM1155957 (GEO)	ATAC-seq	GM12878	None	[4]
	ATAC-seq	K562	None	

GSM1551550, GSM1551551, GSM1551552, GSM1551553, GSM1551554, GSM1551555, GSM1551556, GSM1551557, GSM1551558, GSM1551559, GSM1551560, GSM1551561, GSM1551562, GSM1551563, GSM1551564, GSM1551565, GSM1551566, GSM1551567, GSM1551568, GSM1551569, GSM1551570, GSM1551571, GSM1551572, GSM1551573, GSM1551574,	Hi-C	GM12878	None	[1]

GSM1551575, GSM1551576, GSM1551577, GSM1551578, GSM1551579, GSM1551580, GSM1551581, GSM1551582, GSM1551592, GSM1551592, GSM1551593, GSM1551594, GSM1551595, GSM1551596, GSM1551597, GSM1551598, (GEO)				
GSM1872886 (GEO)	ChIA-PET	GM12878	CTCF	[5]
GSM1436265 (GEO)	ChIA-PET	GM12878	RAD21	[6]
ENCLB784HEF, ENCLB535GER, (ENCODE)	ChIA-PET	GM12878	RAD21	[2]
GSM2138324, GSM2138325, GSM2138326,	HiChIP	GM12878	Cohesin	[7]

GSM2138327 (GEO)				
GSM2705042, GSM2705041, (GEO)	HiChIP	GM12878	H3K27ac	[3]
ERR436029, ERR436028, ERR436030, ERR436033, ERR436031, ERR436026, (ArrayExpress: E-MTAB-2323)	Promoter Capture Hi-C	GM12878	None	[8]
GSM758559 (GEO)	RNA-seq	GM12878	None	[9]
GSM765405 (GEO)	RNA-seq	K562	None	[9]
GSE144336 (GEO)	MCC	E14-mESC	Capturing of Sox2, Klf4 and Myc	[10]
GSE130275 (GEO)	Micro-C	JM8.N4-mESC	None	[11]
GSM1436632 (GEO)	ChIP-seq	E14-mESC	H3K27me3	
GSM1830114 (GEO)	ATAC-seq	E14-mESC	None	[12]
GSM699165	ChIP-seq	E14-mESC	CTCF	[13]

(GEO)				
GSM1014154	DNase-seq	E14-mESC	None	[14]

Processing of published ATAC-seq, ChIA-PET, Hi-C, capture Hi-C and HiChIP data

Raw FASTQ files of ATAC-seq data were mapped to human genome hg38 by Bowtie2 [15]. Peaks were called by MACS2 (v2.1.2) [16].

Raw FASTQ files of ChIA-PET data were pre-processed by Mango [17] to hg38 into unique intra-chromosomal PETs for downstream analysis. GM12878 CTCF ChIA-PET loops were obtained from cLoops [18] (https://github.com/YaqiangCao/cLoops_supplementaryData/tree/master/SupplementaryData/loops/ChIA-PET).

Raw FASTQ files of in situ Hi-C data were pre-processed by HiC-Pro (v2.11.1) [19] to hg38 into unique intra-chromosomal PETs for downstream analysis. GM12878 Hi-C domains and loops were obtained from Juicebox [20] of hg19 and lift over to hg38 for usage.

Raw FASTQ files of capture Hi-C data were pre-processed by HiCUP (v0.7.2) [21] to hg38 into unique intra-chromosomal PETs for downstream analysis. GM12878 capture Hi-C loops were obtained from https://www.ncbi.nlm.nih.gov/geo/query/acc.cgi?acc=GSE81503 of hg19 and converted to hg38 for usage.

Raw FASTQ files of HiChIP data were pre-processed by HiC-Pro (v2.11.1) to hg38 into unique intra-chromosomal PETs for downstream analysis. GM12878 Cohesin HiChIP loops were obtained from cLoops (https://github.com/YaqiangCao/cLoops_supplementaryData/tree/master/SupplementaryData/loops/HiChIP). GM12878 H3K27ac HiChIP loops called by HiCCUPs were obtained from their paper [3] of hg19 and converted to hg38. GM12878 H3K27ac HiChIP loops called by FitHiChIP [22] were obtained from the method paper and converted to hg38. For the overlapping analysis between Hi-TrAC and H3K27ac HiChIP loops, loop anchors were extended to 5 kb, unique and

overlapped loops were obtained by pairtopair subcommand in BEDTools (v2.29.2) package [23] with -type notboth or -type both option.

All above PETs level data were converted to BEDPE format for analysis of reads properties and further processed by the cLoops2 pre module to cLoops2 data directories for visualization and analysis.

Estimation of whole genome-wide interaction resolution

Intra-chromosomal PETs were grouped into contact matrix bins with assigned resolution. Accumulation of PETs (as Y-axis) against the accumulation bins (as X-axis, bins sorted by PETs in the bin ascendingly) for a resolution were plotted to determine the interaction signal enrichment. If all PETs are evenly distributed between genome locations, then there will be a straight diagonal line, which is true if we shuffled the two ends of all PETs to an expected background. Otherwise, if there are specific and strong interactions, PETs will be centralized in only a few bins. Then the curve will show the pattern of prominent and steep rise towards the higher ranked bins. We implemented the idea and checked the lines for different resolutions for Hi-TrAC and other methods. There will be two parts of a signal enrichment curve for interaction data: 1) a straight-line part in lower-ranked bins, which only have singleton PET in each bin. 2) a curve part in higher ranked bins, which contain multiple PETs. There are higher possibilities of those singleton PET bins and PETs being noises/background considering reproducibility. As we assume, there are structures in the interacting 3D genome, then the PETs should have signal enrichment to some degree/resolution. Therefore, we define the highest genome-wide resolution as more than 50% of PETs (solid curves) are in multiple PET bins. The estimation is only a whole-genome wide estimation, and some local regions may have higher or lower resolutions. The idea of this method was initially inspired by fingerprint plots of ChIP-seq quality control implemented in deepTools [24]. This analysis was implemented in cLoops2 as the estRes module.

Genomic regions annotated for Rehoboam plots

RUNX1 of **Fig. 2g.** Regions were obtained from merged loops anchors of Hi-TrAC of both GM12878 and K562 (**Supplementary Table 2**).

Supplementary Table 2: Annotations of regulatory elements of *RUNX1* gene.

chr21	35042620	35055667	Promoter
chr21	34800222	34804065	E0
chr21	34807002	34809347	E1
chr21	34844310	34849154	E2
chr21	34861487	34867811	E3
chr21	34882737	34893033	E4
chr21	34897706	34899137	E5
chr21	34900207	34913168	E6
chr21	34922446	34935524	E7
chr21	34938620	34952208	E8
chr21	34967553	34976280	E9
chr21	34982883	34990637	E10
chr21	34993854	35003932	E11
chr21	35010715	35016764	E12
chr21	35019774	35020584	E13
chr21	35023346	35029770	E14
chr21	35039452	35040565	E15
chr21	35056638	35058010	E16
chr21	35071018	35071807	E17
chr21	35075636	35077527	E18
chr21	35096757	35097907	E19
chr21	35101466	35109883	E20
chr21	35136383	35141973	E21
chr21	35151854	35155775	E22
chr21	35187372	35192749	E23
chr21	35201516	35207016	E24
chr21	35211566	35213423	E25
chr21	35220672	35238567	E26
chr21	35251540	35253635	E27
chr21	35291185	35294263	E28
chr21	35321437	35322042	E29
chr21	35339868	35344138	E30
chr21	35355001	35358292	E31
chr21	35396096	35407470	E32
chr21	35415302	35418536	E33
chr21	35480194	35487455	E34
chr21	35502646	35510875	E35

ZNF gene cluster for **Fig. 5a, b**. Regions were obtained from HCFC1 and ZNF143 overlapped peaks, then extended from the overlapped peaks center for both upstream and downstream 1 kb (**Supplementary Table 3**).

Supplementary Table 3: Annotations of regulatory elements in ZNF gene cluster.

chr19	44001922	44003922	P1
chr19	44024299	44026299	P2
chr19	44050842	44052842	N1
chr19	44071093	44073093	P3
chr19	44093345	44095345	P4
chr19	44112262	44114262	P5
chr19	44140475	44142475	N2
chr19	44164015	44166015	P6
chr19	44206442	44208442	E1
chr19	44211430	44213430	N3
chr19	44258877	44260877	N4
chr19	44281681	44283681	E2
chr19	44304024	44306024	P7

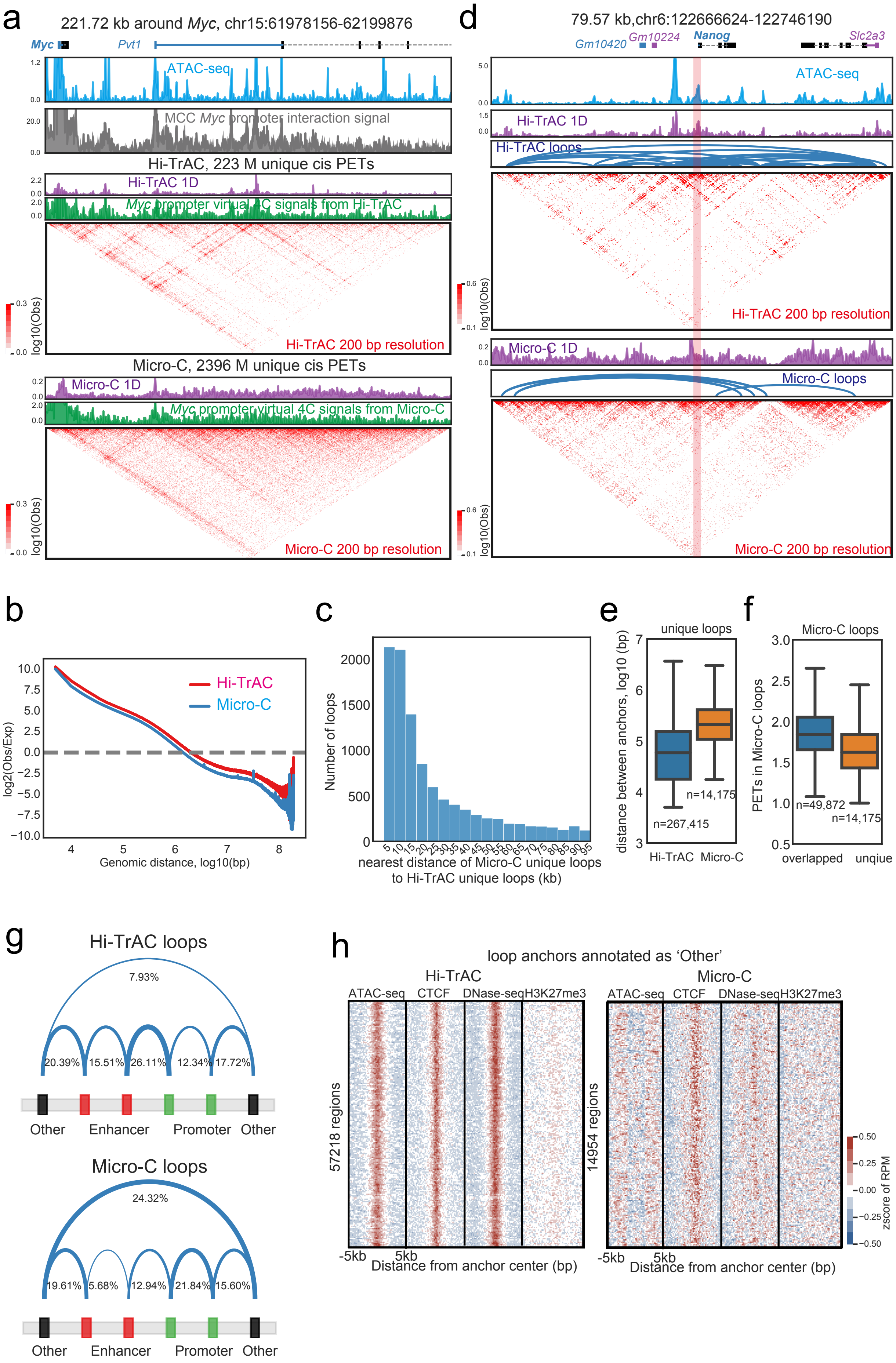

a
221.72 kb around Myc, chr15:61978156-62199876
Myc
Pvt1
ATAC-seq
MCC Myc promoter interaction signal
Hi-TrAC, 223 M unique cis PETs
Hi-TrAC 1D
Myc promoter virtual 4C signals from Hi-TrAC
log10(Obs)
Hi-TrAC 200 bp resolution
Micro-C, 2396 M unique cis PETs
Micro-C 1D
Myc promoter virtual 4C signals from Micro-C
Micro-C 200 bp resolution
d
79.57 kb,chr6:122666624-122746190
Gm10420
Gm10224
Nanog
Slc2a3
ATAC-seq
Hi-TrAC 1D
Hi-TrAC loops
Hi-TrAC 200 bp resolution
Micro-C 1D
Micro-C loops
Micro-C 200 bp resolution
b
Hi-TrAC
Micro-C
log2(Obs/Exp)
Genomic distance, log10(bp)
c
Number of loops
nearest distance of Micro-C unique loops
to Hi-TrAC unique loops (kb)
e
unique loops
distance between anchors, log10 (bp)
n=267,415
n=14,175
Hi-TrAC
Micro-C
f
Micro-C loops
PETs in Micro-C loops
n=49,872
n=14,175
overlapped
unqiue
g
Hi-TrAC loops
7.93%
20.39%
15.51%
26.11%
12.34%
17.72%
Other
Enhancer
Promoter
Other
Micro-C loops
24.32%
19.61%
5.68%
12.94%
21.84%
15.60%
Other
Enhancer
Promoter
Other
h
loop anchors annotated as 'Other'
Hi-TrAC
Micro-C
ATAC-seq
CTCF
DNase-seq
H3K27me3
57218 regions
14954 regions
-5kb
5kb
Distance from anchor center (bp)
zscore of RPM

Supplementary Fig. 1: Comparison of the performance in detecting chromatin loops between Hi-TrAC and Micro-C.

a Comparison of interactions in *Myc* gene and its immediate downstream region. The interaction heatmaps from the Hi-TrAC and Micro-C data were shown at 200 bp resolution. **b** Distribution of interacting PETs frequency against genomic distance for Hi-TrAC and Micro-C. Both axes are log10 transformed. The random shuffling of two ends of the PETs 10 times was used to generate expected background. The analysis was performed with the cLoops2 estDis module. **c** Distance distribution of unique loops from 2.6 billion Micro-C data to unique loops of Hi-TrAC. More than 4,000 (extra ~6%) Micro-C loops are not overlapped with Hi-TrAC loops but are located nearby, which may be caused by some miss alignment of anchors. **d** Chromatin looping profiles at *Nanog* gene locus detected by Hi-TrAC and Micro-C. **e** Distribution of distance between loop anchors for unique loops detected by Hi-TrAC and Micro-C. The box extends from the first quartile to the third quartile of the data, with a line at the median. The whiskers extend from the box by 1.5x the inter-quartile range. Flier points past the end of the whiskers were not shown. n = the number of loops. Source data are provided as a Source Data file. **f** Distribution of the number of PETs for Micro-C overlapped and unique loops with Hi-TrAC. The box extends from the first quartile to the third quartile of the data, with a line at the median. The whiskers extend from the box by 1.5x the inter-quartile range. Flier points past the end of the whiskers were not shown. n = the number of loops. Source data are provided as a Source Data file. **g** The fractions of chromatin loop anchors detected by Hi-TrAC (upper panel) and Micro-C (lower panel), which are located to potential enhancers, promoters, or non-accessible regions. mESC ATAC-seq (GSM1830114[12]) peaks were used to define promoters (peaks within 2 kb of TSS) and enhancers (distance of peaks to TSS > 2kb). “Other” indicates the loop anchor has no overlaps with ATAC-seq peaks. **h** Aggregation analysis of ATAC-seq, DNase-seq, CTCF and H3K27me3 ChIP-seq signals on loop anchors defined as “Other” in panel **g** for Hi-TrAC (left panel) and Micro-C (right panel). These Hi-TrAC anchors are potential weak ATAC-seq peaks missed by peak-calling, meanwhile Micro-C anchors are weak CTCF binding sites.

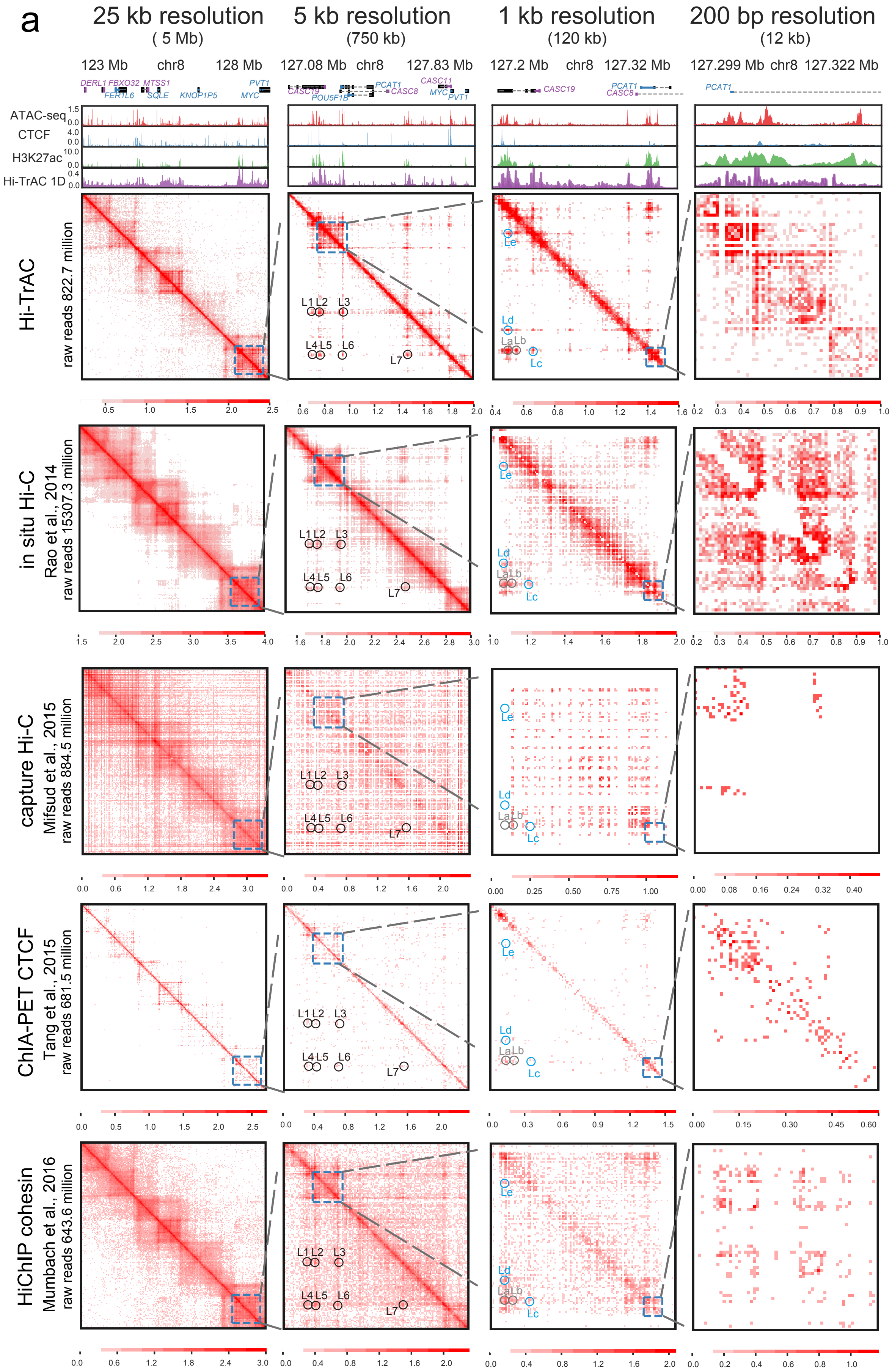
a
25 kb resolution
(5 Mb)
5 kb resolution
(750 kb)
1 kb resolution
(120 kb)
200 bp resolution
(12 kb)
123 Mb chr8 128 Mb
127.08 Mb chr8 127.83 Mb
127.2 Mb chr8 127.32 Mb
127.299 Mb chr8 127.322 Mb
ATAC-seq
CTCF
H3K27ac
Hi-TrAC 1D
Hi-TrAC
raw reads 822.7 million
in situ Hi-C
Rao et al., 2014
raw reads 15307.3 million
capture Hi-C
Mifsud et al., 2015
raw reads 884.5 million
ChIA-PET CTCF
Tang et al., 2015
raw reads 681.5 million
HiChIP cohesin
Mumbach et al., 2016
raw reads 643.6 million
L1 L2 L3
L4 L5 L6
L7
Le
Ld
La Lb
Lc

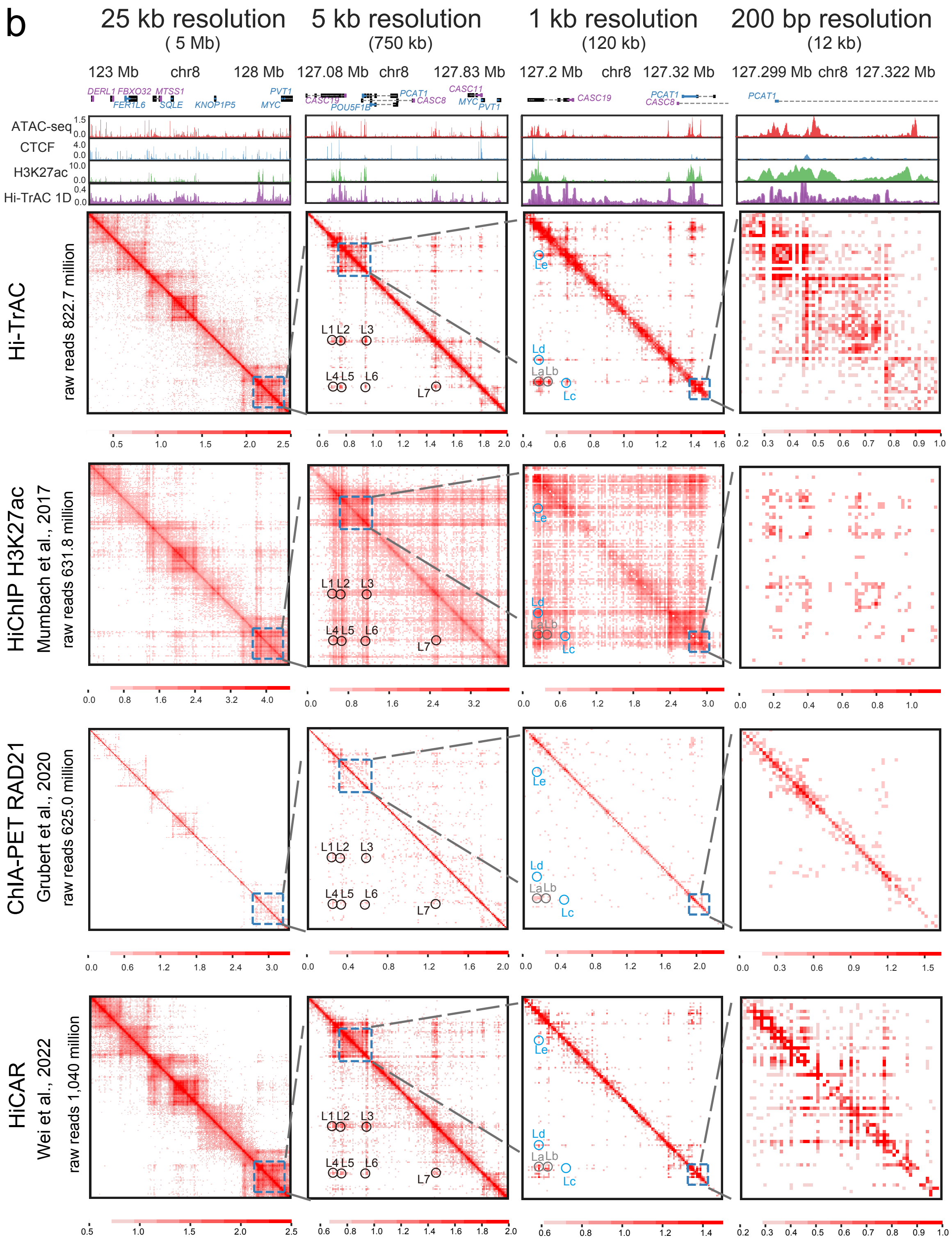

b
25 kb resolution
(5 Mb)
5 kb resolution
(750 kb)
1 kb resolution
(120 kb)
200 bp resolution
(12 kb)
123 Mb chr8 128 Mb
127.08 Mb chr8 127.83 Mb
127.2 Mb chr8 127.32 Mb
127.299 Mb chr8 127.322 Mb
ATAC-seq
CTCF
H3K27ac
Hi-TrAC 1D
Hi-TrAC
raw reads 822.7 million
HiChIP H3K27ac
Mumbach et al., 2017
raw reads 631.8 million
ChIA-PET RAD21
Grubert et al., 2020
raw reads 625.0 million
HiCAR
Wei et al., 2022
raw reads 1,040 million
L1 L2 L3
L4 L5 L6
L7
Le
Ld
La Lb
Lc

Supplementary Fig. 2: Comparison of Hi-TrAC to other techniques for mapping chromatin interactions.

a, b Comparison of chromatin architectures detected by Hi-TrAC and other representative state-of-art chromatin interactions mapping techniques, including in situ Hi-C[25], capture Hi-C[8], CTCF ChIA-PET[26], cohesin HiChIP[7], H3K27ac HiChIP[3], RAD21 ChIA-PET[2], and HiCAR[27] at different resolutions and scales in GM12878 cells around *MYC* gene locus. Numbers of raw sequenced reads were indicated for each method (refer to **Supplementary Data 1** for more details). Log_{10} transformed PETs were shown in the heatmaps. ChIP-seq data were obtained from the ENCODE project[28]. ATAC-seq data were obtained from GSE47753[4]. Visualization was performed with cLoops2 plot module.

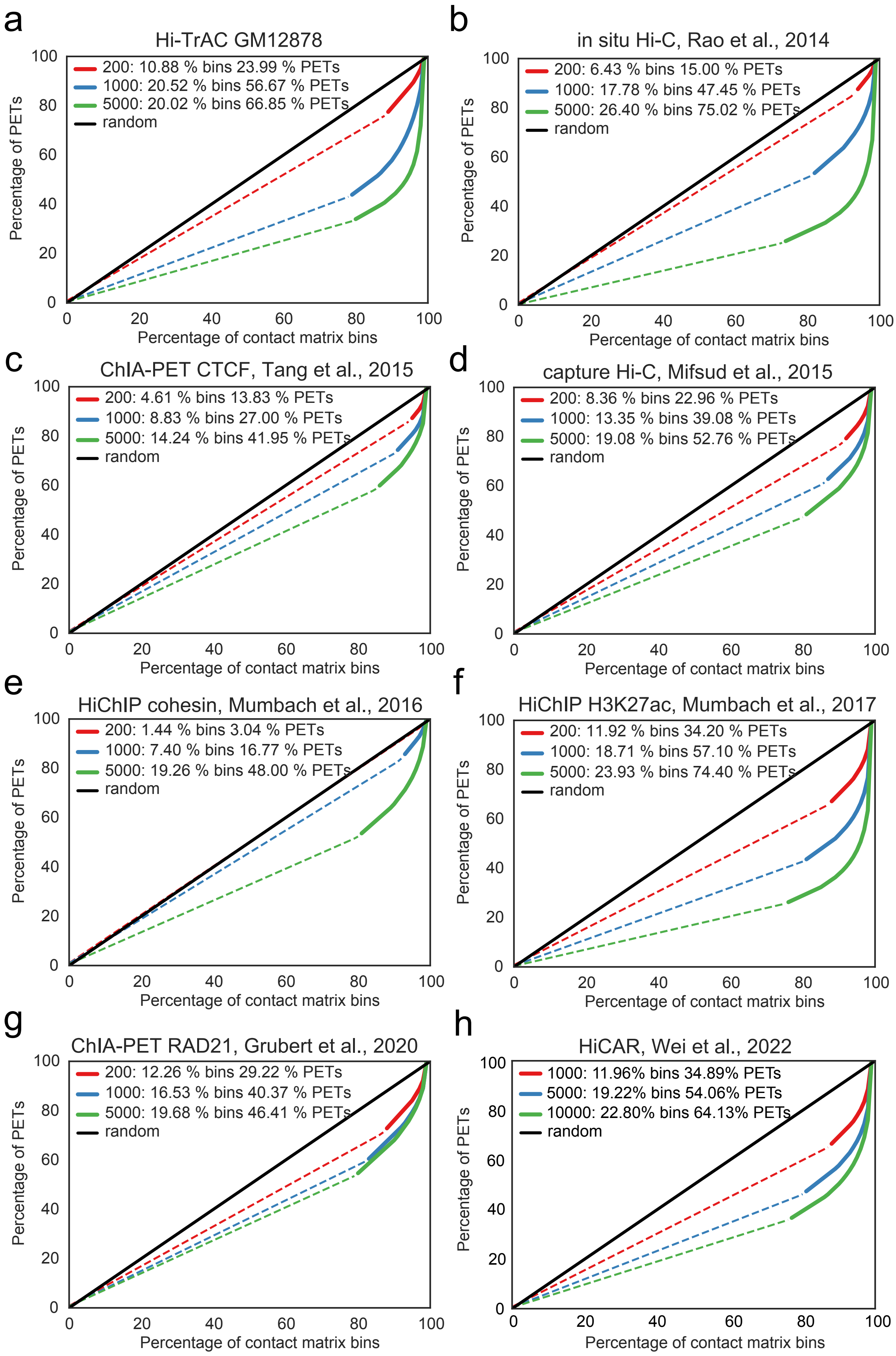

a
Hi-TrAC GM12878
200: 10.88 % bins 23.99 % PETs
1000: 20.52 % bins 56.67 % PETs
5000: 20.02 % bins 66.85 % PETs
random
Percentage of PETs
Percentage of contact matrix bins
b
in situ Hi-C, Rao et al., 2014
200: 6.43 % bins 15.00 % PETs
1000: 17.78 % bins 47.45 % PETs
5000: 26.40 % bins 75.02 % PETs
random
Percentage of PETs
Percentage of contact matrix bins
c
ChIA-PET CTCF, Tang et al., 2015
200: 4.61 % bins 13.83 % PETs
1000: 8.83 % bins 27.00 % PETs
5000: 14.24 % bins 41.95 % PETs
random
Percentage of PETs
Percentage of contact matrix bins
d
capture Hi-C, Mifsud et al., 2015
200: 8.36 % bins 22.96 % PETs
1000: 13.35 % bins 39.08 % PETs
5000: 19.08 % bins 52.76 % PETs
random
Percentage of PETs
Percentage of contact matrix bins
e
HiChIP cohesin, Mumbach et al., 2016
200: 1.44 % bins 3.04 % PETs
1000: 7.40 % bins 16.77 % PETs
5000: 19.26 % bins 48.00 % PETs
random
Percentage of PETs
Percentage of contact matrix bins
f
HiChIP H3K27ac, Mumbach et al., 2017
200: 11.92 % bins 34.20 % PETs
1000: 18.71 % bins 57.10 % PETs
5000: 23.93 % bins 74.40 % PETs
random
Percentage of PETs
Percentage of contact matrix bins
g
ChIA-PET RAD21, Grubert et al., 2020
200: 12.26 % bins 29.22 % PETs
1000: 16.53 % bins 40.37 % PETs
5000: 19.68 % bins 46.41 % PETs
random
Percentage of PETs
Percentage of contact matrix bins
h
HiCAR, Wei et al., 2022
1000: 11.96% bins 34.89% PETs
5000: 19.22% bins 54.06% PETs
10000: 22.80% bins 64.13% PETs
random
Percentage of PETs
Percentage of contact matrix bins

Supplementary Fig. 3: Estimation of resolutions of genome-wide interaction data from different techniques.

a - h Estimation of resolutions for Hi-TrAC, in situ Hi-C, CTCF ChIA-PET, capture Hi-C, cohesin HiChIP, H3K27ac HiChIP, RAD21 ChIA-PET, and HiCAR respectively. The interacting paired-end tags (PETs) were grouped into contact matrix bins based on different resolutions (200bp, 1kb, and 5kb). Dash lines show the contact matrix bins with only singleton PETs, which are evenly distributed and increased linearly and are presumably background noises. Solid curves indicate the bins with multiple PETs. We define the highest genome-wide resolution as more than 50% of PETs (solid curves) are in multiple PET bins. The analysis is implemented in the cLoops2 estRes module.

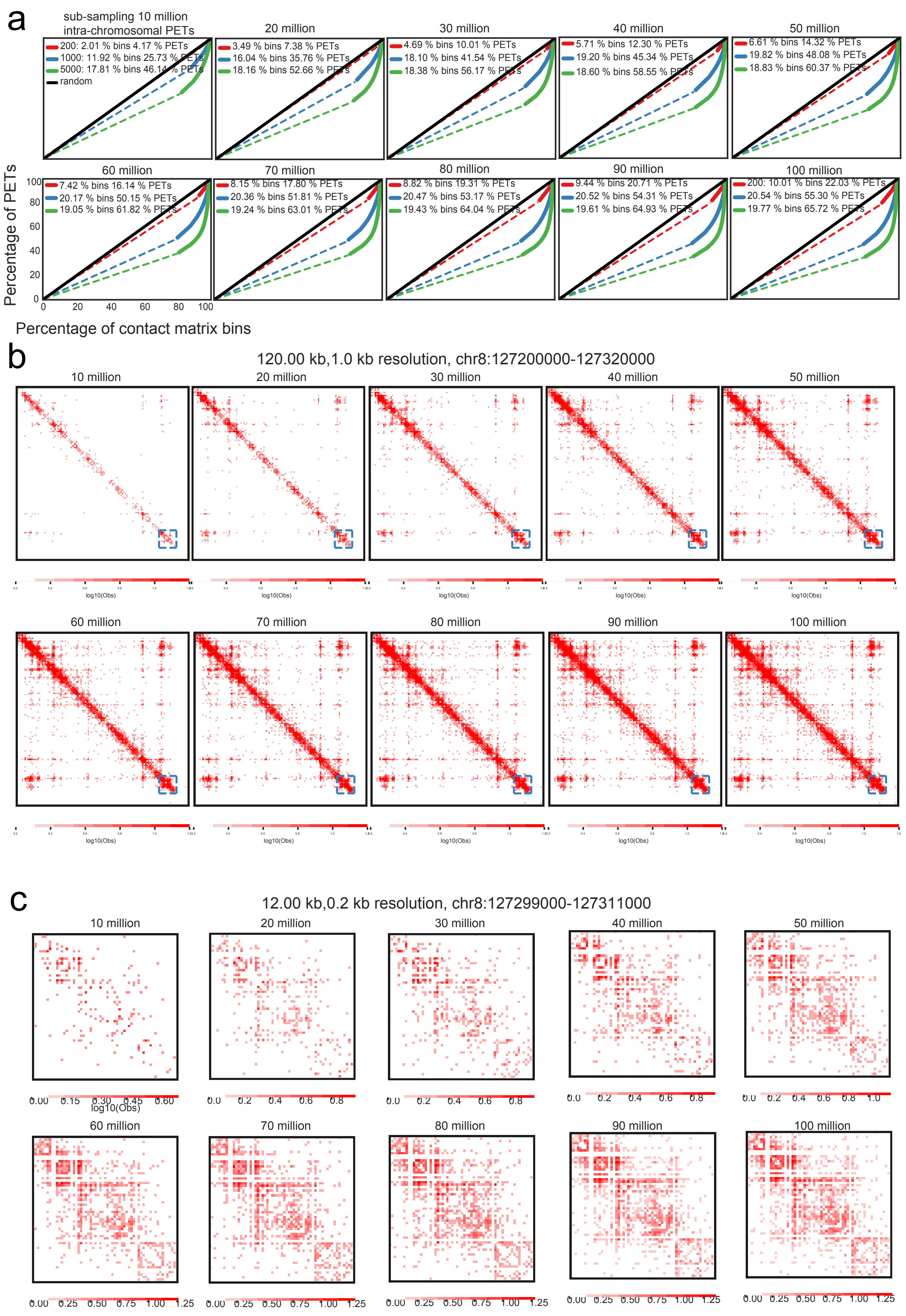
a
sub-sampling 10 million intra-chromosomal PETs
200: 2.01 % bins 4.17 % PETs
1000: 11.92 % bins 25.73 % PETs
5000: 17.81 % bins 46.14 % PETs
random
20 million
3.49 % bins 7.38 % PETs
16.04 % bins 35.76 % PETs
18.16 % bins 52.66 % PETs
30 million
4.69 % bins 10.01 % PETs
18.10 % bins 41.54 % PETs
18.38 % bins 56.17 % PETs
40 million
5.71 % bins 12.30 % PETs
19.20 % bins 45.34 % PETs
18.60 % bins 58.55 % PETs
50 million
6.61 % bins 14.32 % PETs
19.82 % bins 48.08 % PETs
18.83 % bins 60.37 % PETs
60 million
7.42 % bins 16.14 % PETs
20.17 % bins 50.15 % PETs
19.05 % bins 61.82 % PETs
70 million
8.15 % bins 17.80 % PETs
20.36 % bins 51.81 % PETs
19.24 % bins 63.01 % PETs
80 million
8.82 % bins 19.31 % PETs
20.47 % bins 53.17 % PETs
19.43 % bins 64.04 % PETs
90 million
9.44 % bins 20.71 % PETs
20.52 % bins 54.31 % PETs
19.61 % bins 64.93 % PETs
100 million
200: 10.01 % bins 22.03 % PETs
20.54 % bins 55.30 % PETs
19.77 % bins 65.72 % PETs
Percentage of PETs
Percentage of contact matrix bins
b
120.00 kb,1.0 kb resolution, chr8:127200000-127320000
10 million
20 million
30 million
40 million
50 million
60 million
70 million
80 million
90 million
100 million
c
12.00 kb,0.2 kb resolution, chr8:127299000-127311000
10 million
20 million
30 million
40 million
50 million
log10(Obs)
60 million
70 million
80 million
90 million
100 million

Supplementary Fig. 4: Downsampling of GM12878 Hi-TrAC data for estimating the required sequencing depth to achieve desired resolution.

a Estimation of interaction resolutions with different sub-sampling depths of final unique intra-chromosomal PETs. With 60 million final unique intra-chromosomal PETs, Hi-TrAC can achieve the resolution of 1 kb. **b** The interaction matrix heatmaps for the example region shown in **Supplementary Fig. 2** with 1 kb resolution at different sub-sampling depth. **c** The interaction matrix heatmaps for the super-enhancer region shown in **Supplementary Fig. 2** at 200 bp resolution. With 50 million final unique intra-chromosomal PETs, Hi-TrAC can detect clear sub-structures of the super-enhancer.

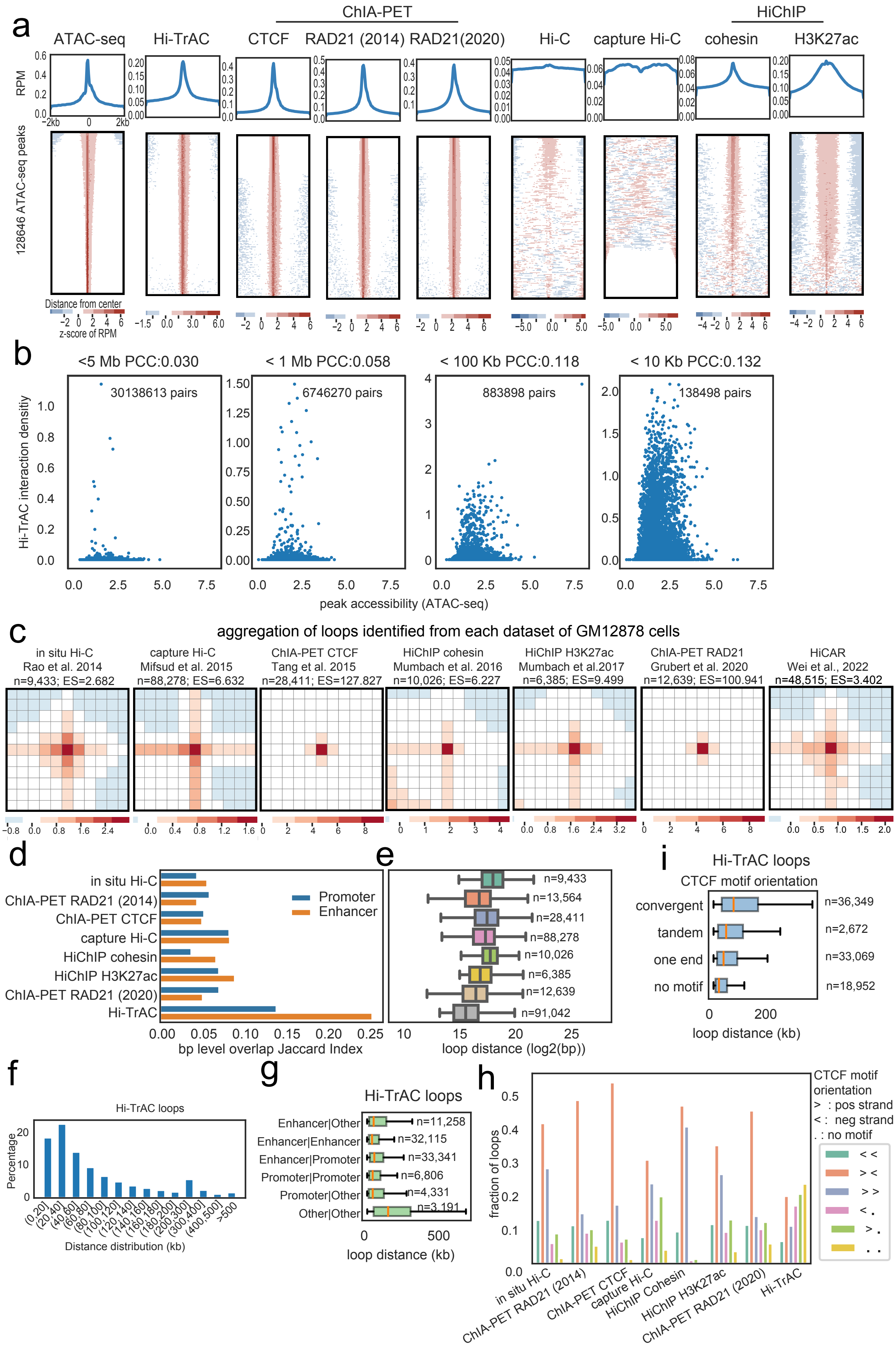
a
ChIA-PET
HiChIP
ATAC-seq
Hi-TrAC
CTCF
RAD21 (2014)
RAD21(2020)
Hi-C
capture Hi-C
cohesin
H3K27ac
RPM
128646 ATAC-seq peaks
Distance from center
z-score of RPM
b
<5 Mb PCC:0.030
30138613 pairs
< 1 Mb PCC:0.058
6746270 pairs
< 100 Kb PCC:0.118
883898 pairs
< 10 Kb PCC:0.132
138498 pairs
Hi-TrAC interaction density
peak accessibility (ATAC-seq)
c
aggregation of loops identified from each dataset of GM12878 cells
in situ Hi-C
Rao et al. 2014
n=9,433; ES=2.682
capture Hi-C
Mifsud et al. 2015
n=88,278; ES=6.632
ChIA-PET CTCF
Tang et al. 2015
n=28,411; ES=127.827
HiChIP cohesin
Mumbach et al. 2016
n=10,026; ES=6.227
HiChIP H3K27ac
Mumbach et al.2017
n=6,385; ES=9.499
ChIA-PET RAD21
Grubert et al. 2020
n=12,639; ES=100.941
HiCAR
Wei et al., 2022
n=48,515; ES=3.402
d
in situ Hi-C
ChIA-PET RAD21 (2014)
ChIA-PET CTCF
capture Hi-C
HiChIP cohesin
HiChIP H3K27ac
ChIA-PET RAD21 (2020)
Hi-TrAC
Promoter
Enhancer
bp level overlap Jaccard Index
e
n=9,433
n=13,564
n=28,411
n=88,278
n=10,026
n=6,385
n=12,639
n=91,042
loop distance (log2(bp))
i
Hi-TrAC loops
CTCF motif orientation
convergent
tandem
one end
no motif
n=36,349
n=2,672
n=33,069
n=18,952
loop distance (kb)
f
Hi-TrAC loops
Percentage
(0,20]
(20,40]
(40,60]
(60,80]
(80,100]
(100,120]
(120,140]
(140,160]
(160,180]
(180,200]
(200,300]
(300,400]
(400,500]
>500
Distance distribution (kb)
g
Hi-TrAC loops
Enhancer|Other
Enhancer|Enhancer
Enhancer|Promoter
Promoter|Promoter
Promoter|Other
Other|Other
n=11,258
n=32,115
n=33,341
n=6,806
n=4,331
n=3,191
loop distance (kb)
h
fraction of loops
CTCF motif
orientation
> : pos strand
< : neg strand
. : no motif
< <
> <
> >
< .
> .
. .
in situ Hi-C
ChIA-PET RAD21 (2014)
ChIA-PET CTCF
capture Hi-C
HiChIP Cohesin
HiChIP H3K27ac
ChIA-PET RAD21 (2020)
Hi-TrAC

Supplementary Fig. 5: Features of chromatin loops detected by Hi-TrAC and other techniques.

a Aggregation plots of 1D signals measured by Hi-TrAC and other 3D techniques at ATAC-seq peaks. **b** Genome-wide correlation analysis between Hi-TrAC detected interaction density and ATAC-seq detected accessibility. Briefly, ATAC-seq peaks within the linear genomic distance of 5 Mb, 1 Mb, 100 kb, and 10 kb were combined exhaustively to measure the accessibility and count the Hi-TrAC interacting PETs. For two peaks marked as x and y, integrated accessibility is measured as $\sqrt{r_x \times r_y}$, where r_x or r_y is the ATAC-seq signal quantified into RPKM for peak x or y (normalized by peak length). The interaction density is measured as $P_{x,y}/(W_x + W_y)$, where $P_{x,y}$ is the number of PETs linking peaks x and y from Hi-TrAC data and W_x or W_y is the peak width of x or y. **c** Aggregation analysis of loops identified by other techniques. The numbers of loops called from each dataset are indicated. **d** Overlaps of loop anchors and cis-regulatory elements at base-pair level. A higher Jaccard index indicates anchor and enhancer/promoter matches with more similar size. **e** Distribution of anchor distance of loops detected by different techniques. The box extends from the first quartile to the third quartile of the data, with a line at the median. The whiskers extend from the box by 1.5x the inter-quartile range. Flier points past the end of the whiskers were not shown. n = the number of loops. Source data are provided as a Source Data file. **f** Distance distribution of GM12878 Hi-TrAC loop anchors. Most of the loops are formed by anchors within 200 kb. **g** Distance distribution of GM12878 loop anchors classified by cis-regulatory elements. The box extends from the first quartile to the third quartile of the data, with a line at the median. The whiskers extend from the box by 1.5x the inter-quartile range. Flier points past the end of the whiskers were not shown. n = the number of loops. Source data are provided as a Source Data file. **h** Summary of loop compositions with regard to CTCF motif orientation of two anchors. **i** Distance distribution of GM12878 loop anchors classified by CTCF motif orientation combinations. The box extends from the first quartile to the third quartile of the data, with a line at the median. The whiskers extend from the box by 1.5x the inter-quartile range. Flier points past the end of the whiskers were not shown. n = the number of loops. Source data are provided as a Source Data file.

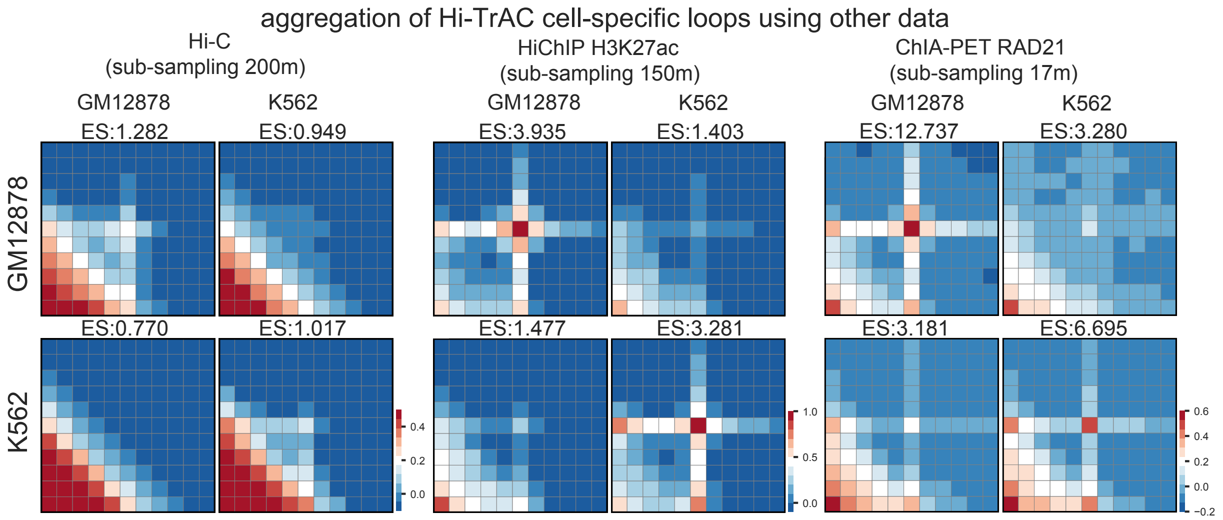

Supplementary Fig. 6: Validation of Hi-TrAC identified cell-specific loops.

Aggregated loops analysis of Hi-TrAC cell-specific loops (**Supplementary Data 4**) using in situ Hi-C, HiChIP H3K27ac, and RAD21 ChIA-PET data. Enrichment score (ES) is calculated as the loop signal (the number of PETs at the matrix center) divided by nearby background (mean of the rest of the matrix except the center). ES > 1 indicates loops have relatively more interactions than nearby regions. A higher difference of ES indicates more differences of interacting PETs in the loops.

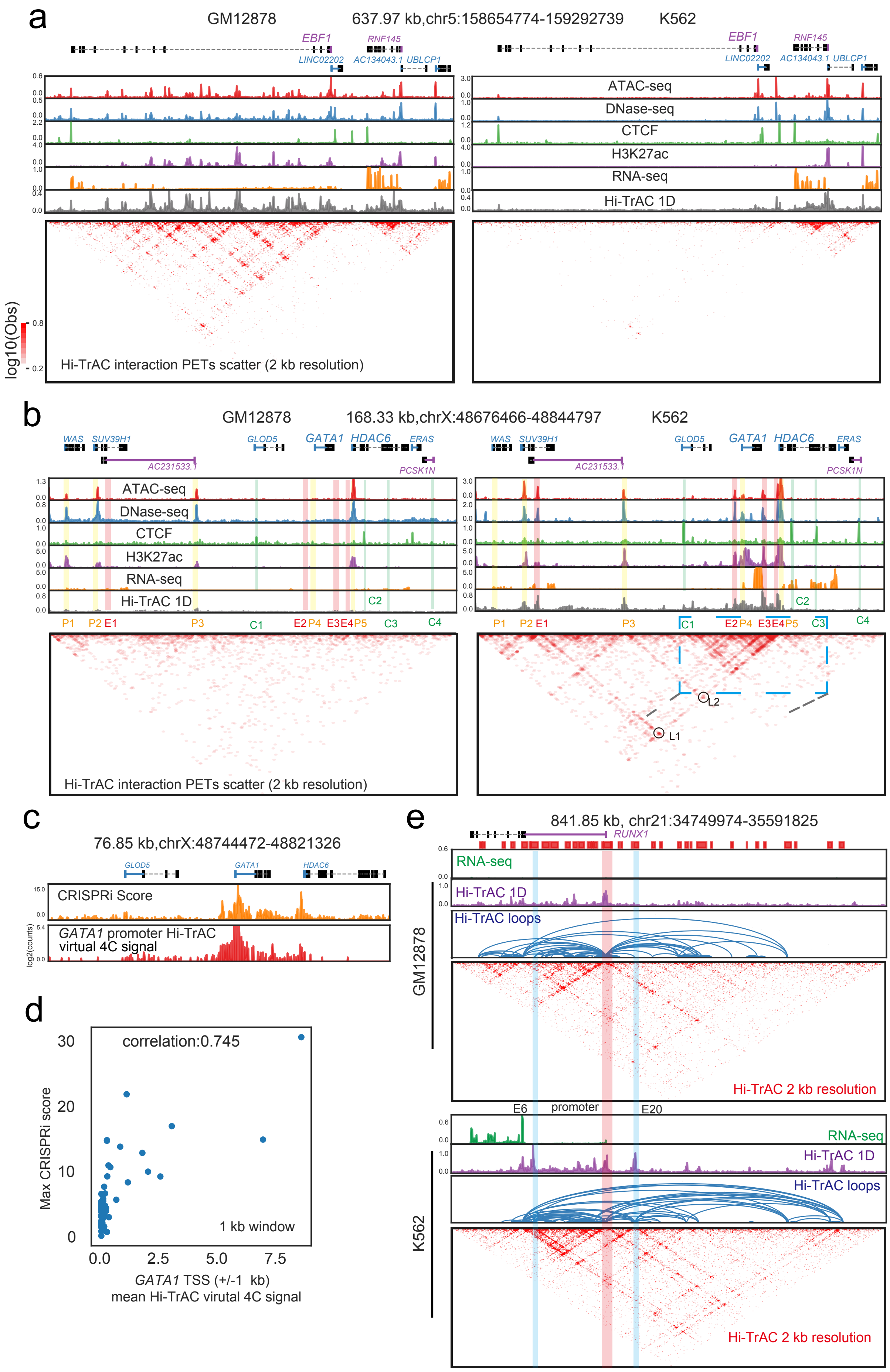
a
GM12878
637.97 kb,chr5:158654774-159292739
K562
EBF1
RNF145
LINC02202
AC134043.1
UBLCP1
ATAC-seq
DNase-seq
CTCF
H3K27ac
RNA-seq
Hi-TrAC 1D
log10(Obs)
Hi-TrAC interaction PETs scatter (2 kb resolution)
b
GM12878
168.33 kb,chrX:48676466-48844797
K562
WAS
SUV39H1
AC231533.1
GLOD5
GATA1
HDAC6
ERAS
PCSK1N
P1 P2 E1 P3 C1 E2 P4 E3 E4 P5 C2 C3 C4
L1
L2
Hi-TrAC interaction PETs scatter (2 kb resolution)
c
76.85 kb,chrX:48744472-48821326
CRISPRi Score
GATA1 promoter Hi-TrAC virtual 4C signal
log2(counts)
d
correlation:0.745
1 kb window
Max CRISPRi score
GATA1 TSS (+/-1 kb)
mean Hi-TrAC virutal 4C signal
e
841.85 kb, chr21:34749974-35591825
RUNX1
RNA-seq
Hi-TrAC 1D
Hi-TrAC loops
GM12878
Hi-TrAC 2 kb resolution
E6
promoter
E20
K562

Supplementary Fig. 7: Hi-TrAC detects cell-specific interactions among regulatory elements at *EBF1*, *GATA1* and *RUNX1* gene loci.

a The chromatin interaction profiles of *EBF1* gene locus detected by Hi-TrAC in GM12878 and K562 cells. ATAC-seq, DNase-seq, CTCF and H3K27ac ChIP-seq, and RNA-seq profiles are shown below the genomic annotations on the top, and interaction matrices are shown at the bottom. **b** The chromatin interaction profiles of *GATA1* gene locus detected by Hi-TrAC in GM12878 and K562 cells. Putative promoters were marked as P1 to P5, putative enhancers were marked as E1 to E4, and CTCF binding sites were marked as C1 to C4. The blue box region was highlighted for zoom-in presentation in panel **c**, and this region was studied before with CRISPR interference to identify regulatory elements of *GATA1* gene[29]. **c** Comparison of Hi-TrAC virtual 4C signals from *GATA1* promoter with CRISPRi scores reported previously[29]. **d** Correlation analysis of CRISPRi scores and Hi-TrAC virtual 4C signals from *GATA1* promoter. **e** Genome Browser snapshots showing looping profiles in GM12878 and K562 cells detected by Hi-TrAC at *RUNX1* gene locus.

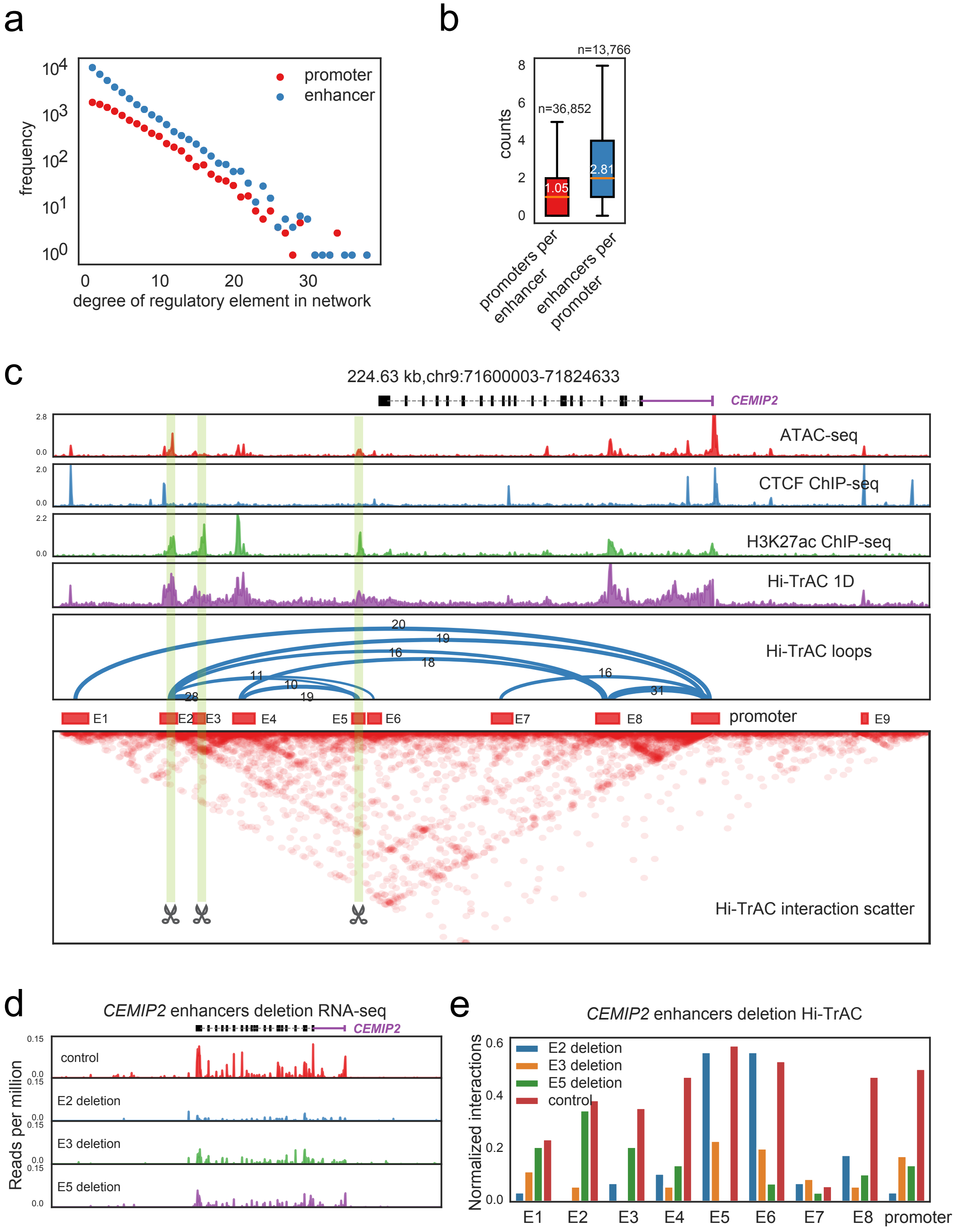

a
frequency
promoter
enhancer
degree of regulatory element in network
b
counts
n=36,852
n=13,766
1.05
2.81
promoters per enhancer
enhancers per promoter
c
224.63 kb,chr9:71600003-71824633
CEMIP2
ATAC-seq
CTCF ChIP-seq
H3K27ac ChIP-seq
Hi-TrAC 1D
Hi-TrAC loops
E1
E2
E3
E4
E5
E6
E7
E8
promoter
E9
Hi-TrAC interaction scatter
d
CEMIP2 enhancers deletion RNA-seq
CEMIP2
Reads per million
control
E2 deletion
E3 deletion
E5 deletion
e
CEMIP2 enhancers deletion Hi-TrAC
Normalized interactions
E2 deletion
E3 deletion
E5 deletion
control
E1
E2
E3
E4
E5
E6
E7
E8
promoter

Supplementary Fig. 8: Regulation of gene expression by the promoter-enhancer interaction network.

a Connection degree distributions of enhancers and promoters in the regulatory network constructed from GM12878 Hi-TrAC loops follow the scale-free network power-law. **b** Distributions of the numbers of looped targets per promoter or per enhancer. The box extends from the first quartile to the third quartile of the data, with a line at the median. The whiskers extend from the box by 1.5x the inter-quartile range. Flier points past the end of the whiskers were not shown. n = the number of enhancers or promoters. Source data are provided as a Source Data file. **c** Loops detected by Hi-TrAC for *CEMIP2* gene in K562 cells. Putative enhancers marked as E2, E3, and E5 were selected for deleting by CRISPR/Cas9. **d** RNA-seq assays showed decreased expression of *CEMIP2* by deleting E2, E3, or E5 as indicated in panel **c**. *ZNF234* promoter on a different chromosome was deleted as the negative control. **e** Interactions of *CEMIP2* promoter and enhancers decreased after the deletion of the putative enhancer E2, E3, or E5. The interactions were measured with Hi-TrAC data.

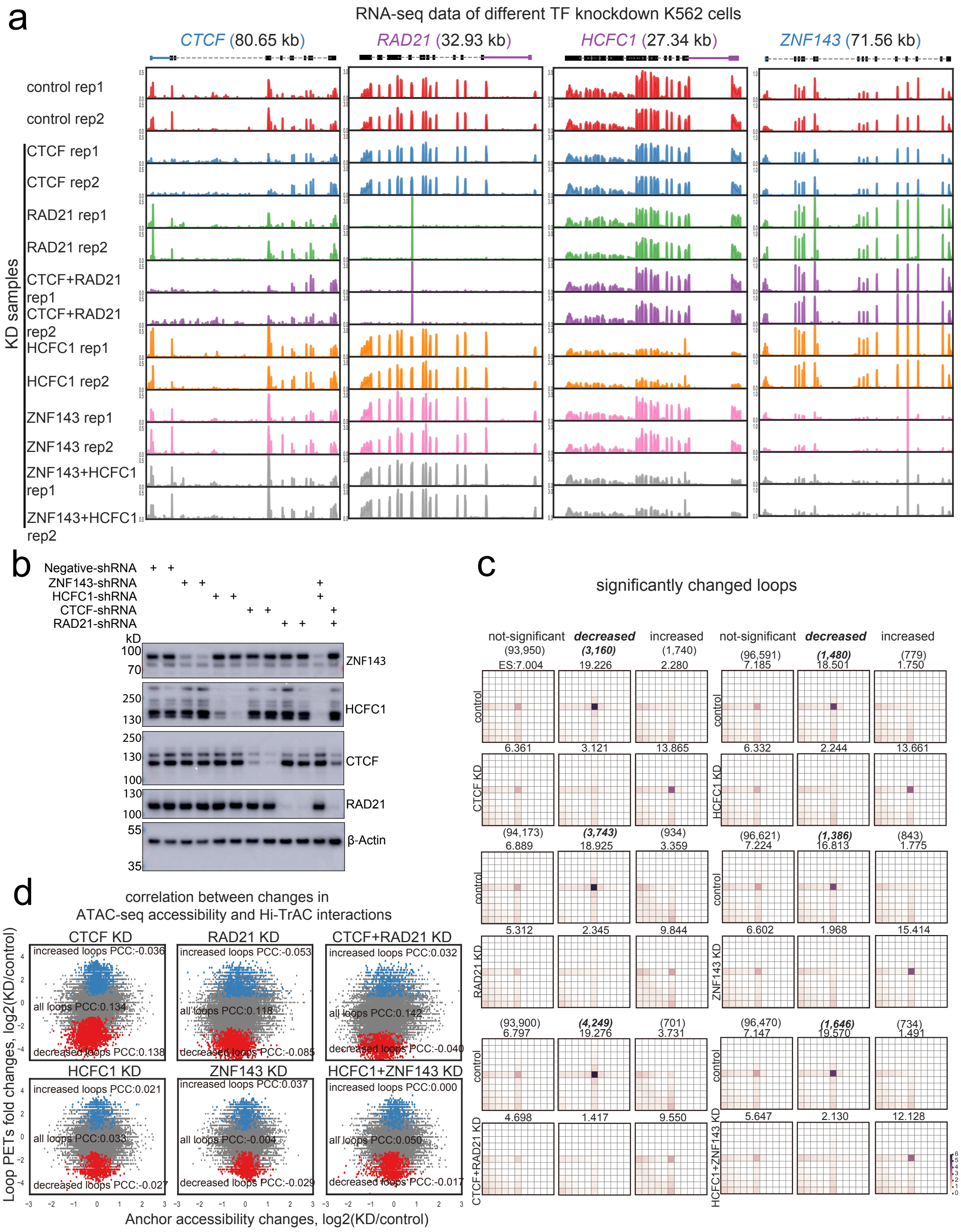
a
RNA-seq data of different TF knockdown K562 cells
CTCF (80.65 kb)
RAD21 (32.93 kb)
HCFC1 (27.34 kb)
ZNF143 (71.56 kb)
control rep1
control rep2
KD samples
CTCF rep1
CTCF rep2
RAD21 rep1
RAD21 rep2
CTCF+RAD21 rep1
CTCF+RAD21 rep2
HCFC1 rep1
HCFC1 rep2
ZNF143 rep1
ZNF143 rep2
ZNF143+HCFC1 rep1
ZNF143+HCFC1 rep2
b
Negative-shRNA
ZNF143-shRNA
HCFC1-shRNA
CTCF-shRNA
RAD21-shRNA
kD
ZNF143
HCFC1
CTCF
RAD21
β-Actin
c
significantly changed loops
not-significant
decreased
increased
control
CTCF KD
HCFC1 KD
RAD21 KD
ZNF143 KD
CTCF+RAD21 KD
HCFC1+ZNF143 KD
d
correlation between changes in ATAC-seq accessibility and Hi-TrAC interactions
CTCF KD
RAD21 KD
CTCF+RAD21 KD
HCFC1 KD
ZNF143 KD
HCFC1+ZNF143 KD
increased loops PCC:-0.036
all loops PCC:0.134
decreased loops PCC:0.138
increased loops PCC:-0.053
all loops PCC:0.118
decreased loops PCC:-0.085
increased loops PCC:0.032
all loops PCC:0.142
decreased loops PCC:-0.040
increased loops PCC:0.021
all loops PCC:0.033
decreased loops PCC:-0.027
increased loops PCC:0.037
all loops PCC:-0.004
decreased loops PCC:-0.029
increased loops PCC:0.000
all loops PCC:0.050
decreased loops PCC:-0.017
Loop PETs fold changes, log2(KD/control)
Anchor accessibility changes, log2(KD/control)

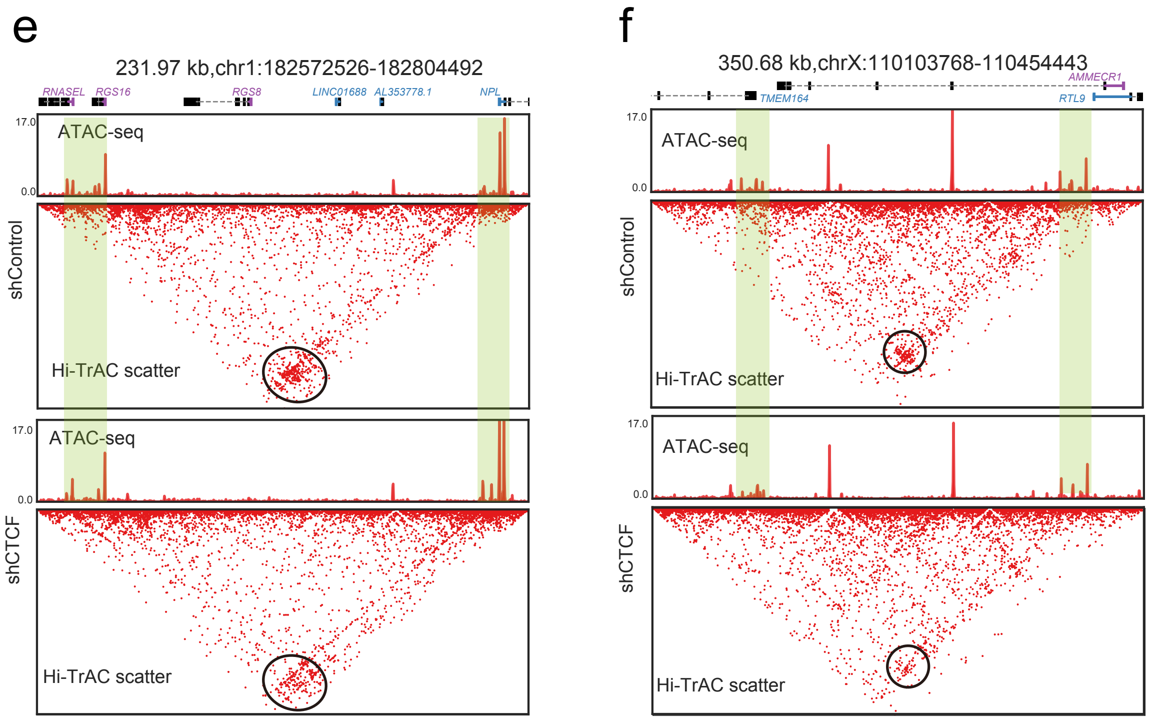

Supplementary Fig. 9: Knocking down ZNF143, HCFC1, CTCF, and RAD21 compromised chromatin looping in K562 cells.

a K562 cells were infected with lentivirus carrying shRNA targeting either ZNF143, HCFC1, CTCF, or RAD21 alone or in combinations. The transcription of target genes were assessed by RNA-seq. **b** The expression of ZNF143, HCFC1, CTCF and RAD21 in knockdown cells were examined by western blotting. **c** Aggregation analysis of differentially enriched loops in knockdown cells (**Supplementary Data 6**). **d** Correlation analysis between the changes in accessibility measured by ATAC-seq and the changes in interactions measured by Hi-TrAC. **e** Genome Browser screenshots of a randomly picked example showing not decreased accessibility of anchors, but decreased interaction. **f** Genome Browser screenshots of another randomly picked example showing not decreased accessibility of anchors, but decreased interaction.

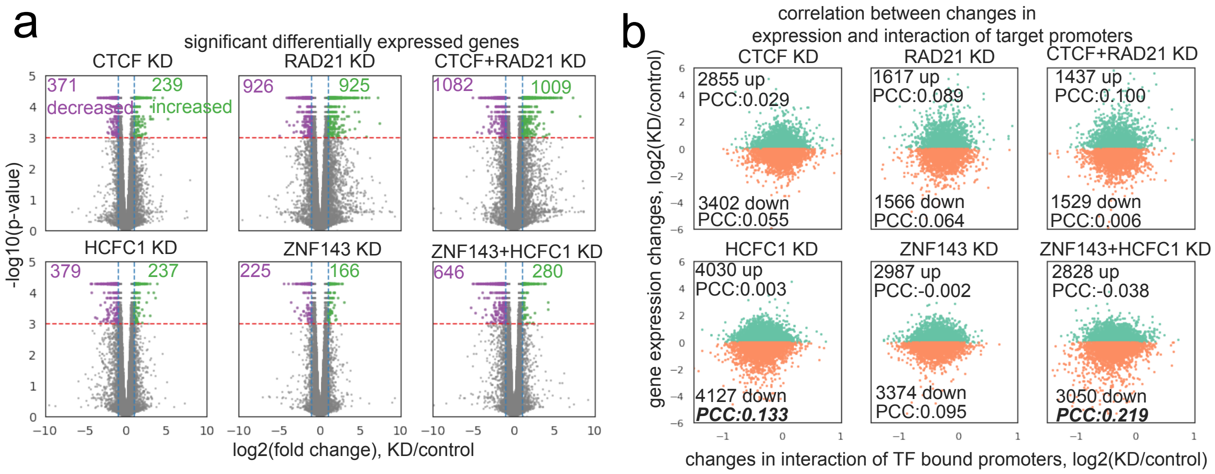

Supplementary Fig. 10: Gene expression impaired by knocking down CTCF, RAD21, HCFC1, and ZNF143.

a Volcano plots showing significantly differentially expressed genes after knocking down CTCF, RAD21, HCFC1, and ZNF143 either alone or in combination. Purple dots indicate genes with decreased expression and green dots indicate genes with increased expression. The P-values were drawn from Cuffdiff output. **b** Correlation analysis between the changes in interactions at promoters and changes in gene expression. Interaction changes from Hi-TrAC data were measured for genes with promoters (+/- 1Kb of TSS) overlapping with loop anchors bound by the targeted TF. PCC stands for Pearson Correlation Coefficient.

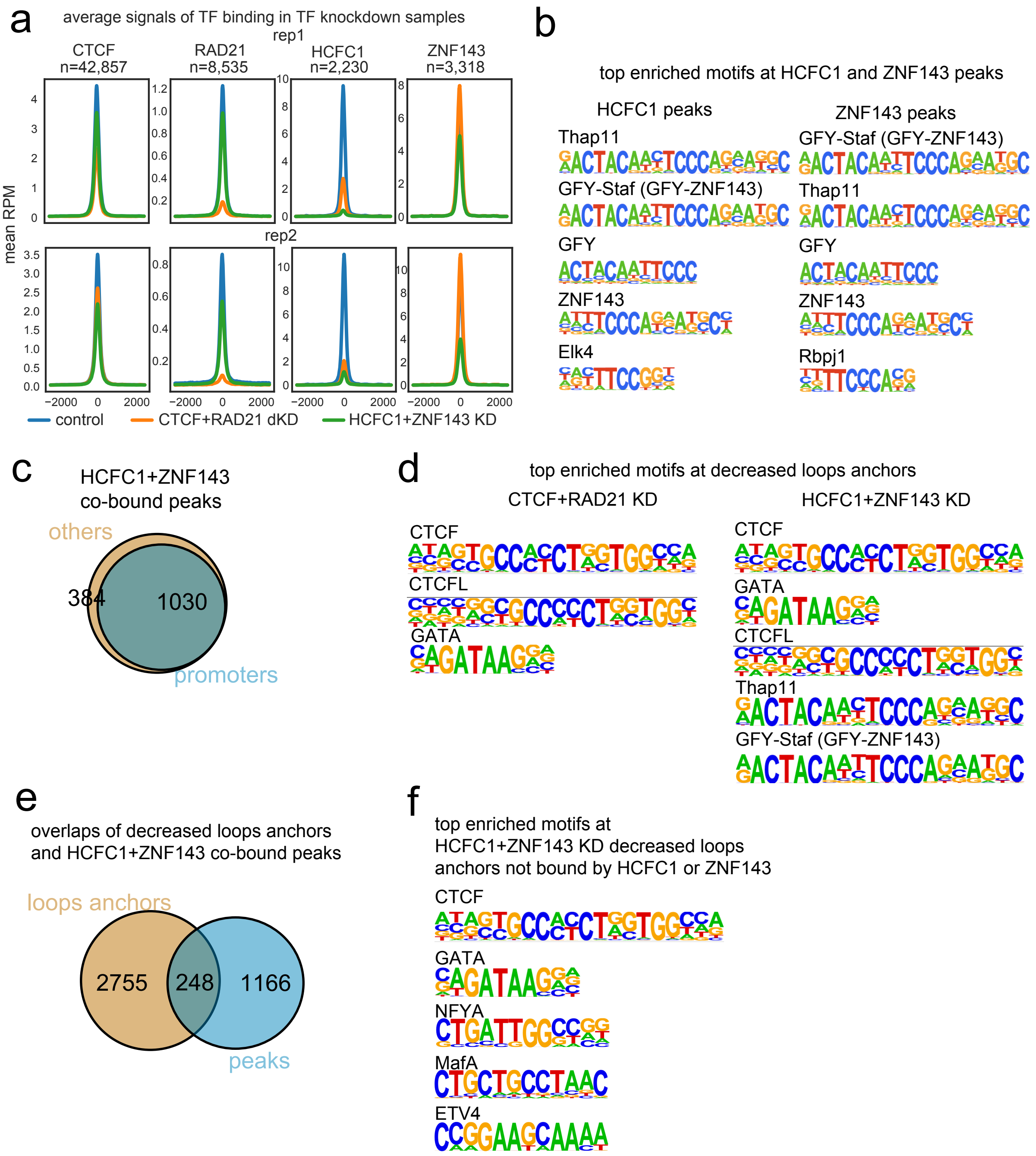
a
average signals of TF binding in TF knockdown samples
rep1
CTCF n=42,857
RAD21 n=8,535
HCFC1 n=2,230
ZNF143 n=3,318
rep2
mean RPM
−2000 0 2000
control
CTCF+RAD21 dKD
HCFC1+ZNF143 KD
b
top enriched motifs at HCFC1 and ZNF143 peaks
HCFC1 peaks
Thap11
GFY-Staf (GFY-ZNF143)
GFY
ZNF143
Elk4
ZNF143 peaks
GFY-Staf (GFY-ZNF143)
Thap11
GFY
ZNF143
Rbpj1
c
HCFC1+ZNF143 co-bound peaks
others
384
1030
promoters
d
top enriched motifs at decreased loops anchors
CTCF+RAD21 KD
CTCF
CTCFL
GATA
HCFC1+ZNF143 KD
CTCF
GATA
CTCFL
Thap11
GFY-Staf (GFY-ZNF143)
e
overlaps of decreased loops anchors and HCFC1+ZNF143 co-bound peaks
loops anchors
2755
248
1166
peaks
f
top enriched motifs at HCFC1+ZNF143 KD decreased loops anchors not bound by HCFC1 or ZNF143
CTCF
GATA
NFYA
MafA
ETV4

Supplementary Fig. 11: Characterization of loop anchors bound by different transcription factors.

a Aggregated profile analysis for the changes of CTCF, RAD21, HCFC1 and ZNF143 binding after knocking down CTCF with RAD21, or HCFC1 with ZNF143. Overlapped peaks from control samples were used for the analysis. **b** Top enriched motifs found by HOMER from HCFC1 or ZNF143 peaks identified in K562 shRNA control cells. **c** Overlaps of HCFC1 and ZNF143 co-bound peaks and gene promoters. **d** Top enriched motifs found by HOMER from decreased loop anchors detected by Hi-TrAC in CTCF and RAD21 double knockdown (left panel) or HCFC1 and ZNF143 double knockdown (right panel) cells. **e** Overlaps of HCFC1 and ZNF143 co-bound peaks with significantly decreased loop anchors called from Hi-TrAC data after knocking down of HCFC1 and ZNF143. **f** Top enriched motifs in decreased loop anchors not bound by HCFC1 or ZNF143 in the HCFC1 and ZNF143 double knockdown cells.

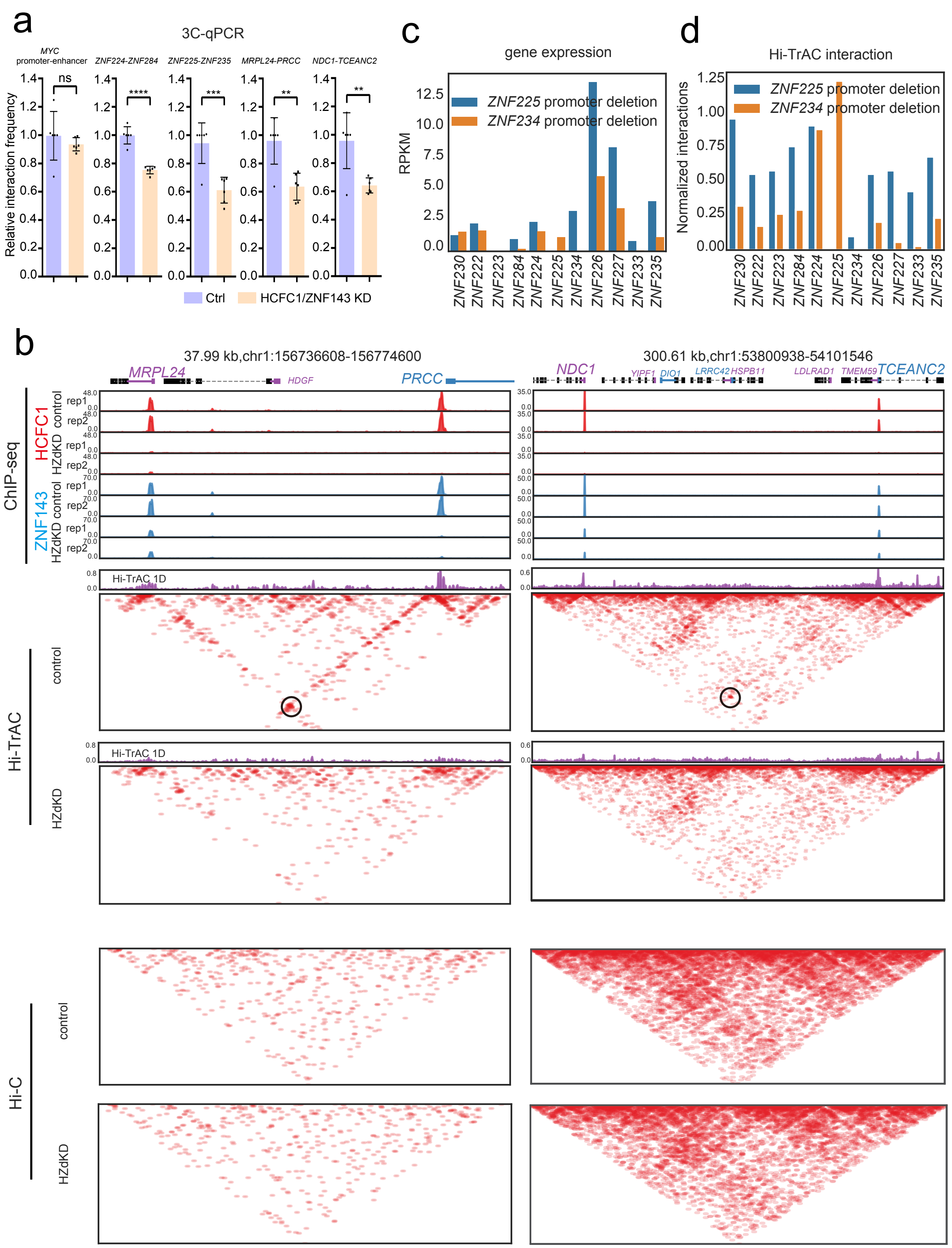

a
3C-qPCR
MYC promoter-enhancer
ZNF224-ZNF284
ZNF225-ZNF235
MRPL24-PRCC
NDC1-TCEANC2
Relative interaction frequency
ns
****
***
**
**
Ctrl
HCFC1/ZNF143 KD
c
gene expression
RPKM
ZNF225 promoter deletion
ZNF234 promoter deletion
d
Hi-TrAC interaction
Normalized interactions
b
37.99 kb,chr1:156736608-156774600
300.61 kb,chr1:53800938-54101546
MRPL24
HDGF
PRCC
NDC1
YIPF1
DIO1
LRRC42
HSPB11
LDLRAD1
TMEM59
TCEANC2
ChIP-seq
HCFC1
ZNF143
control
HZdKD
rep1
rep2
Hi-TrAC
Hi-TrAC 1D
Hi-C

Supplementary Fig. 12. HCFC1 and ZNF143 associated promoter-promoter loops regulate gene expression.

a 3C-qPCR quantitative analysis of the interaction frequency changes of *ZNF224-ZNF284*, *ZNF225-ZNF235*, *MRPL24-PRCC* and *NDC1-TCEANC2* in HCFC1 and ZNF143 knockdown cells. A promoter-enhancer loop of *MYC* gene was included as negative control. The abundance of these interaction pairs in 3C libraries were quantified by qPCR, and normalized to input control (promoter of *MYC* gene). The columns represent mean ± s.d., n = 6 (three independent experiments, each experiment has two technical replicates), *MYC* promoter-enhancer p = 0.4295, *ZNF224-ZNF284* p < 0.0001, *ZNF225-ZNF235* p = 0.0007, *MRPL24-PRCC* p = 0.0019, and *NDC1-TCEANC2* p = 0.0036 according to two-sided *t*-test. ns not significant, **** p < 0.0001, *** p < 0.001, ** p < 0.01. Source data are provided as a Source Data file. **b** Genome Browser snapshots showing *MRPL24-PRCC* and *NDC1-TCEANC2* loops impaired by knocking down HCFC1 and ZNF143 as detected by Hi-TrAC. The binding patterns of HCFC1 and ZNF143 (ChIP-seq tracks) at these regions are also presented. **c** The expression change of ZNF genes after deleting *ZNF225* or *ZNF234* promoter. **d** The chromatin interaction change of ZNF gene promoters after deleting *ZNF225* or *ZNF234* promoter.

Reference

1 Rao, Suhas S. P. *et al.* A 3D Map of the Human Genome at Kilobase Resolution Reveals Principles of Chromatin Looping. *Cell* **159**, 1665-1680, doi:10.1016/j.cell.2014.11.021 (2014).

2 Grubert, F. *et al.* Landscape of cohesin-mediated chromatin loops in the human genome. *Nature* **583**, 737-743, doi:10.1038/s41586-020-2151-x (2020).

3 Mumbach, M. R. *et al.* Enhancer connectome in primary human cells identifies target genes of disease-associated DNA elements. *Nat Genet* **49**, 1602-1612, doi:10.1038/ng.3963 (2017).

4 Buenrostro, J. D., Giresi, P. G., Zaba, L. C., Chang, H. Y. & Greenleaf, W. J. Transposition of native chromatin for fast and sensitive epigenomic profiling of open chromatin, DNA-binding proteins and nucleosome position. *Nat Methods* **10**, 1213-1218, doi:10.1038/nmeth.2688 (2013).

5 Tang, Z. *et al.* CTCF-Mediated Human 3D Genome Architecture Reveals Chromatin Topology for Transcription. *Cell* **163**, 1611-1627, doi:http://dx.doi.org/10.1016/j.cell.2015.11.024 (2015).

6 Heidari, N. *et al.* Genome-wide map of regulatory interactions in the human genome. *Genome Res* **24**, 1905-1917, doi:10.1101/gr.176586.114 (2014).

7 Mumbach, M. R. *et al.* HiChIP: efficient and sensitive analysis of protein-directed genome architecture. *Nat Methods* **13**, 919-922, doi:10.1038/nmeth.3999 (2016).

8 Mifsud, B. *et al.* Mapping long-range promoter contacts in human cells with high-resolution capture Hi-C. *Nat Genet* **47**, 598-606, doi:10.1038/ng.3286 (2015).

9 Djebali, S. *et al.* Landscape of transcription in human cells. *Nature* **489**, 101-108, doi:10.1038/nature11233 (2012).

10 Hua, P. *et al.* Defining genome architecture at base-pair resolution. *Nature* **595**, 125-129, doi:10.1038/s41586-021-03639-4 (2021).

11 Hsieh, T. S. *et al.* Resolving the 3D Landscape of Transcription-Linked Mammalian Chromatin Folding. *Mol Cell*, doi:10.1016/j.molcel.2020.03.002 (2020).

12 Di Stefano, B. *et al.* C/EBPalpha creates elite cells for iPSC reprogramming by upregulating Klf4 and increasing the levels of Lsd1 and Brd4. *Nat Cell Biol* **18**, 371-381, doi:10.1038/ncb3326 (2016).

13 Handoko, L. *et al.* CTCF-mediated functional chromatin interactome in pluripotent cells. *Nat Genet* **43**, 630-638, doi:10.1038/ng.857 (2011).

14 Yue, F. *et al.* A comparative encyclopedia of DNA elements in the mouse genome. *Nature* **515**, 355-364, doi:10.1038/nature13992 (2014).

15 Langmead, B. & Salzberg, S. L. Fast gapped-read alignment with Bowtie 2. *Nature Methods* **9**, 357-U354, doi:10.1038/Nmeth.1923 (2012).

16 Zhang, Y. *et al.* Model-based analysis of ChIP-Seq (MACS). *Genome Biol* **9**, R137, doi:10.1186/gb-2008-9-9-r137 (2008).

17 Phanstiel, D. H., Boyle, A. P., Heidari, N. & Snyder, M. P. Mango: a bias-correcting ChIA-PET analysis pipeline. *Bioinformatics* **31**, 3092-3098, doi:10.1093/bioinformatics/btv336 (2015).

18 Cao, Y. *et al.* Accurate loop calling for 3D genomic data with cLoops. *Bioinformatics* **36**, 666-675, doi:10.1093/bioinformatics/btz651 (2020).

19 Servant, N. *et al.* HiC-Pro: an optimized and flexible pipeline for Hi-C data processing. *Genome Biol* **16**, 259, doi:10.1186/s13059-015-0831-x (2015).
20 Durand, N. C. *et al.* Juicebox Provides a Visualization System for Hi-C Contact Maps with Unlimited Zoom. *Cell Syst* **3**, 99-101, doi:10.1016/j.cels.2015.07.012 (2016).
21 Wingett, S. *et al.* HiCUP: pipeline for mapping and processing Hi-C data. *F1000Res* **4**, 1310, doi:10.12688/f1000research.7334.1 (2015).
22 Bhattacharyya, S., Chandra, V., Vijayanand, P. & Ay, F. Identification of significant chromatin contacts from HiChIP data by FitHiChIP. *Nat Commun* **10**, 4221, doi:10.1038/s41467-019-11950-y (2019).
23 Quinlan, A. R. & Hall, I. M. BEDTools: a flexible suite of utilities for comparing genomic features. *Bioinformatics* **26**, 841-842, doi:10.1093/bioinformatics/btq033 (2010).
24 Ramirez, F. *et al.* deepTools2: a next generation web server for deep-sequencing data analysis. *Nucleic Acids Res* **44**, W160-165, doi:10.1093/nar/gkw257 (2016).
25 Rao, S. S. *et al.* A 3D map of the human genome at kilobase resolution reveals principles of chromatin looping. *Cell* **159**, 1665-1680, doi:10.1016/j.cell.2014.11.021 (2014).
26 Tang, Z. *et al.* CTCF-Mediated Human 3D Genome Architecture Reveals Chromatin Topology for Transcription. *Cell* **163**, 1611-1627, doi:10.1016/j.cell.2015.11.024 (2015).
27 Wei, X. *et al.* HiCAR is a robust and sensitive method to analyze open-chromatin-associated genome organization. *Mol Cell* **82**, 1225-1238 e1226, doi:10.1016/j.molcel.2022.01.023 (2022).
28 Consortium, E. P. An integrated encyclopedia of DNA elements in the human genome. *Nature* **489**, 57-74, doi:10.1038/nature11247 (2012).
29 Fulco, C. P. *et al.* Systematic mapping of functional enhancer-promoter connections with CRISPR interference. *Science* **354**, 769-773, doi:10.1126/science.aag2445 (2016).
