# Supplementary material for: Hi-TrAC reveals division of labor of transcription factors in organizing chromatin loops
Source: Nat Commun. 2022 Nov 5;13:6679. doi: 10.1038/s41467-022-34276-8 (PMC9637178; doi:10.1038/s41467-022-34276-8)
Supplement: Supplementary file 2 — Description of Additional Supplementary Files [file 41467_2022_34276_MOESM2_ESM.pdf]

Description of Additional Supplementary Files

File Name: Supplementary Data 1
Description: Summary of Hi-TrAC reads generated in this study.

File Name: Supplementary Data 2
Description: Hi-TrAC loops identified in mESCs.

File Name: Supplementary Data 3
Description: Hi-TrAC loops identified in GM12878 and K562 cells.

File Name: Supplementary Data 4
Description: GM12878 and K562 cell-specific loops.

File Name: Supplementary Data 5
Description: GM12878 and K562 Hi-TrAC loops associated transcription factors.

File Name: Supplementary Data 6
Description: Differentially enriched Hi-TrAC loops for K562 shRNA knocking down samples.

File Name: Supplementary Data 7
Description: Summary of ATAC-seq reads and peaks for K562 shRNA knocking down samples.

File Name: Supplementary Data 8
Description: Summary of Hi-C reads for K562 shRNA knocking down samples.

File Name: Supplementary Data 9
Description: Gene expression profiles of K562 shRNA knocking down samples.

File Name: Supplementary Data 10
Description: Summary of ChIP-seq reads and peaks for K562 shRNA knocking down samples.

File Name: Supplementary Data 11
Description: Effect of linker length on Hi-TrAC data.
