# Supplementary material for: Hi-TrAC reveals division of labor of transcription factors in organizing chromatin loops
Source: Nat Commun. 2022 Nov 5;13:6679. doi: 10.1038/s41467-022-34276-8 (PMC9637178; doi:10.1038/s41467-022-34276-8)
Supplement: Supplementary file 14 — Reporting Summary [file 41467_2022_34276_MOESM14_ESM.pdf]

nature portfolio

Corresponding author(s): Keji Zhao

Last updated by author(s): Oct 11, 2022

Reporting Summary

Nature Portfolio wishes to improve the reproducibility of the work that we publish. This form provides structure for consistency and transparency in reporting. For further information on Nature Portfolio policies, see our Editorial Policies and the Editorial Policy Checklist.

Statistics

For all statistical analyses, confirm that the following items are present in the figure legend, table legend, main text, or Methods section.

n/a	Confirmed	
☐	☒	The exact sample size (*n*) for each experimental group/condition, given as a discrete number and unit of measurement
☐	☒	A statement on whether measurements were taken from distinct samples or whether the same sample was measured repeatedly
☐	☒	The statistical test(s) used AND whether they are one- or two-sided<br>*Only common tests should be described solely by name; describe more complex techniques in the Methods section.*
☒	☐	A description of all covariates tested
☐	☒	A description of any assumptions or corrections, such as tests of normality and adjustment for multiple comparisons
☐	☒	A full description of the statistical parameters including central tendency (e.g. means) or other basic estimates (e.g. regression coefficient) AND variation (e.g. standard deviation) or associated estimates of uncertainty (e.g. confidence intervals)
☐	☒	For null hypothesis testing, the test statistic (e.g. *F*, *t*, *r*) with confidence intervals, effect sizes, degrees of freedom and *P* value noted<br>*Give P values as exact values whenever suitable.*
☒	☐	For Bayesian analysis, information on the choice of priors and Markov chain Monte Carlo settings
☒	☐	For hierarchical and complex designs, identification of the appropriate level for tests and full reporting of outcomes
☐	☒	Estimates of effect sizes (e.g. Cohen's *d*, Pearson's *r*), indicating how they were calculated

Our web collection on statistics for biologists contains articles on many of the points above.

Software and code

Policy information about availability of computer code

Data collection	wget (v1.20.3) in Linux was used for downloading data from GEO, ENCODE or ArrayExpress database. Re-analyzed public data were summarized in Supplemental Methods.
Data analysis	cLoops2 (v0.0.1): https://github.com/YaqiangCao/cLoops2<br>Bowtie2 (v2.3.5): http://bowtie-bio.sourceforge.net/bowtie2/index.shtml<br>STAR (v2.7.3a): https://github.com/alexdobin/STAR<br>Cufflinks (v2.2.1): http://cole-trapnell-lab.github.io/cufflinks/<br>MACS2 (v2.1.2): https://pypi.org/project/MACS2<br>Mango (v1.2.0): https://github.com/dphansti/mango<br>HiCUP (v0.7.2): https://www.bioinformatics.babraham.ac.uk/projects/hicup<br>HiC-Pro (v2.11.1): https://github.com/nservant/HiC-Pro<br>deepTools (v3.3.0): https://deeptools.readthedocs.io/en/develop/index.html<br>FIMO (v5.0.2): https://meme-suite.org/meme/doc/fimo.html<br>HOMER (v4.11.1): http://homer.ucsd.edu/homer/index.html<br>matplotlib (v3.2.2): https://matplotlib.org/<br>seaborn (v0.11.0):https://seaborn.pydata.org/<br>scikit-learn (v0.23.2):https://scikit-learn.org/stable/<br>TensorFlow (v1.10.0):https://github.com/tensorflow/tensorflow<br>NetworkX (v2.4): https://networkx.org/<br>Usage of these softwares and other custom analysis were fully described in the manuscript.

For manuscripts utilizing custom algorithms or software that are central to the research but not yet described in published literature, software must be made available to editors and reviewers. We strongly encourage code deposition in a community repository (e.g. GitHub). See the Nature Portfolio guidelines for submitting code & software for further information.

Data

Policy information about availability of data

All manuscripts must include a data availability statement. This statement should provide the following information, where applicable:

- Accession codes, unique identifiers, or web links for publicly available datasets
- A description of any restrictions on data availability
- For clinical datasets or third party data, please ensure that the statement adheres to our policy

Hi-TrAC, RNA-seq, ATAC-seq, Hi-C and ChIP-seq data generated by this study have been deposited to GEO with public accession of GSE180175.

Field-specific reporting

Please select the one below that is the best fit for your research. If you are not sure, read the appropriate sections before making your selection.

☒ Life sciences ☐ Behavioural & social sciences ☐ Ecological, evolutionary & environmental sciences

For a reference copy of the document with all sections, see nature.com/documents/nr-reporting-summary-flat.pdf

Life sciences study design

All studies must disclose on these points even when the disclosure is negative.

Sample size	All the experiments were performed with well established cell-lines in this study, and the number of cells used in each experiment was sufficient. The number of cells used in the experiments was determined using a cell counter. 0.1 or 1 million cells were used for each Hi-TrAC experiment in human cells. 0.01, 0.1 or 1 million cells were used for each Hi-TrAC experiment in mouse cells. 5,000 cells were used for each RNA-seq experiment. 0.1 million cells were used for CTCF and RAD21 ChIP-seq experiments, and 0.5 million cells were used for HCFC1 and ZNF143 ChIP-seq experiments. 50,000 cells were used for each ATAC-seq experiment. 1 million cells were used for each Hi-C experiment.
Data exclusions	No data were excluded for analysis.
Replication	All the experiments in this study were performed with at least two biological replicates and three technical replicates. The data quality of each replicate was confirmed. There are two biological replicates and three technical replicates of Hi-TrAC data for wild type K562 and GM12878 cells. There are two biological replicates of Hi-TrAC data and RNA-seq data for 4 transcription factors and control knocking down K562 samples. There are two biological replicates of ChIP-seq data for 4 transcription factors knocking down K562 samples. There are two biological replicates of ATAC-seq data for four transcription factors knocking down K562 samples. There are four technical replicates Hi-TrAC data for E14 mESC cells from 1 million cells, 100,000 cells and 10,000 cells. There are two technical replicates Hi-TrAC data fro R1 mESC cells from 100,000 cells and 10,000 cells.
Randomization	All the experiments were performed with well established cell-lines in this study. No randomization was needed for data collection. Some presentation examples were randomly selected.
Blinding	The data in this study were processed and analyzed with computer programs. No blinding was required in this study. The investigators were not blinded during data collection and analysis.

Reporting for specific materials, systems and methods

We require information from authors about some types of materials, experimental systems and methods used in many studies. Here, indicate whether each material, system or method listed is relevant to your study. If you are not sure if a list item applies to your research, read the appropriate section before selecting a response.

Materials & experimental systems

n/a	Involved in the study	
☐	☒	Antibodies
☐	☒	Eukaryotic cell lines
☒	☐	Palaeontology and archaeology
☒	☐	Animals and other organisms
☒	☐	Human research participants
☒	☐	Clinical data
☒	☐	Dual use research of concern

Methods

n/a	Involved in the study	
☐	☒	ChIP-seq
☒	☐	Flow cytometry
☒	☐	MRI-based neuroimaging

Antibodies

Antibodies used	anti-CTCF (Cell Signaling Technology, 3418S), anti-RAD21 (Abcam, ab217678), anti-HCFC1 (Santa Cruz Biotechnology, sc-390950),

Antibodies used | anti-HCFC1 (Cell Signaling Technology, 69690S) and anti-ZNF143 (Abnova Corporation, H00007702-M01). The dilution and amount of antibodies used in the experiments were described in the Methods section of the manuscript.

Validation | The antibodies used in this study were selected based on their extensive use in literatures, which could be found on the product description page of supplier's website.

Eukaryotic cell lines

Policy information about cell lines

Cell line source(s): GM12878 cells were purchased from Coriell Institute; K562 (CCL-243), 293T (CRL-3216), mES-E14TG2a (CRL-1821), and mES-R1 (SCRC-1011) cells were purchased from ATCC.

Authentication: No authentication was performed.

Mycoplasma contamination: The cell lines were not tested for Mycoplasma contamination.

Commonly misidentified lines (See ICLAC register): No commonly misidentified lines were used in this study.

ChIP-seq

Data deposition

☒ Confirm that both raw and final processed data have been deposited in a public database such as GEO.

☒ Confirm that you have deposited or provided access to graph files (e.g. BED files) for the called peaks.

Data access links
May remain private before publication.
Hi-TrAC, RNA-seq, ChIP-seq, ATAC-seq and Hi-C data generated by this study have been deposited to GEO with public accession of GSE180175.

Files in database submission:
BigWig files for downloading and visualization. Raw fastq files are available corresponding to the GSM accession.
GSM5454729_ChIP-seq_control_input.bw
GSM5454730_ChIP-seq_CRdKD_input.bw
GSM5454731_ChIP-seq_HZdKD_input.bw
GSM5454732_ChIP-seq_control_CTCF.bw
GSM5454733_ChIP-seq_control_HCFC1.bw
GSM5454734_ChIP-seq_control_RAD21.bw
GSM5454735_ChIP-seq_control_ZNF143.bw
GSM5454736_ChIP-seq_CRdKD_CTCF.bw
GSM5454737_ChIP-seq_CRdKD_HCFC1.bw
GSM5454738_ChIP-seq_CRdKD_RAD21.bw
GSM5454739_ChIP-seq_CRdKD_ZNF143.bw
GSM5454740_ChIP-seq_HZdKD_CTCF.bw
GSM5454741_ChIP-seq_HZdKD_HCFC1.bw
GSM5454742_ChIP-seq_HZdKD_RAD21.bw
GSM5454743_ChIP-seq_HZdKD_ZNF143.bw
GSM6042012_control_CTCF_rep2.bw
GSM6042013_control_HCFC1_rep2.bw
GSM6042014_control_RAD21_rep2.bw
GSM6042015_control_ZNF143_rep2.bw
GSM6042016_CRdKD_CTCF_rep2.bw
GSM6042017_CRdKD_HCFC1_rep2.bw
GSM6042018_CRdKD_RAD21_rep2.bw
GSM6042019_CRdKD_ZNF143_rep2.bw
GSM6042020_HZdKD_CTCF_rep2.bw
GSM6042021_HZdKD_HCFC1_rep2.bw
GSM6042022_HZdKD_RAD21_rep2.bw
GSM6042023_HZdKD_ZNF143_rep2.bw

Genome browser session (e.g. UCSC): WashU Epigenome Browser (http://epigenomegateway.wustl.edu/browser/) session bundle id: 0faadf10-ba71-11ec-bbb1-e31d6a961339

Methodology

Replicates: Two biological replicates were performed.

Sequencing depth:
50 bp paired-end sequencing were performed. Sequencing depth, unique mapping ratio are listed as following.
sample TotalReads MappingRatio(%s) totalMappedPETs uniquePETs
control_input 36334158 96.91 31088132 28515358
CRdKD_input 38386100 96.64 32555021 29932090
HZdKD_input 34245389 97 29214905 26831165
control_CTCF_rep1 26624531 76.81 18443870 13653836

control_HCFC1_rep1 57368957 10.35 5278462 2734129
control_RAD21_rep1 22907700 90.64 18460777 15400610
control_ZNF143_rep1 55264233 51.16 25049659 16055486
CRdKD_CTCF_rep1 28064847 29.03 7319951 4890256
CRdKD_HCFC1_rep1 44003084 19.96 7823374 4727947
CRdKD_RAD21_rep1 39525487 87.87 30878265 24407184
CRdKD_ZNF143_rep1 32934928 46.76 13701434 8945192
HZdKD_CTCF_rep1 23629024 74.86 15917976 11356761
HZdKD_HCFC1_rep1 46813775 15.21 6337528 3859275
HZdKD_RAD21_rep1 22993491 83.48 17104633 14489345
HZdKD_ZNF143_rep1 43297106 19.61 7579508 4702294
control_CTCF_rep2 30149655 78.13 21187235 15640273
control_HCFC1_rep2 55726748 8.14 4034817 2017738
control_RAD21_rep2 21860691 83.32 16162956 14122602
control_ZNF143_rep2 38404473 40.24 13698286 7942769
CRdKD_CTCF_rep2 36504423 43.41 14245996 7352067
CRdKD_HCFC1_rep2 55073195 22.59 11043283 5918434
CRdKD_RAD21_rep2 35441649 87.18 27319702 24379294
CRdKD_ZNF143_rep2 27415813 29.43 7162867 4025340
HZdKD_CTCF_rep2 25966587 84.66 19726823 16779002
HZdKD_HCFC1_rep2 52472257 10.53 4889146 2933871
HZdKD_RAD21_rep2 27526840 90.61 22132634 19611995
HZdKD_ZNF143_rep2 45871080 26.36 10797534 6807628

Antibodies

anti-CTCF (Cell Signaling Technology, 3418S), anti-RAD21 (Abcam, ab217678), anti-HCFC1 (Cell Signaling Technology, 69690S) and anti-ZNF143 (Abnova Corporation, H00007702-M01)

Peak calling parameters

Raw ChIP-seq data reads were mapped to human reference genome hg38 by Bowtie2 with key parameters of --local --very-sensitive --no-unal --no-mixed --no-discordant. Mapped PETs with MAPQ >=10 were converted to normalized signals (reads per million) as bigWig files by deepTools for visualization. Peaks were called by cLoops2 with key parameters of -eps 150 -minPts 5,10 and input sample set as -bgd.

Data quality

There are more than 91% of CTCF peaks, 56% of RAD21 peaks, 99% of HCFC1 peaks and 78% of ZNF143 peaks have more than 5 folds signal enrichment comparing to input samples. Motifs were checked for all peaks and all 4 TFs enriched known motifs. Visualization through genome browser also confirmed strong binding peaks.

Software

cLoops2 (v0.0.1): https://github.com/YaqiangCao/cLoops2
Bowtie2 (v2.3.5): http://bowtie-bio.sourceforge.net/bowtie2/index.shtml
deepTools (v3.3.0): https://deeptools.readthedocs.io/en/develop/index.html
